# Magnetically driven formation of 3D freestanding soft bioscaffolds

**DOI:** 10.1126/sciadv.adl1549

**Published:** 2024-02-02

**Authors:** Ruoxiao Xie, Yuanxiong Cao, Rujie Sun, Richard Wang, Alexis Morgan, Junyoung Kim, Sebastien J. P. Callens, Kai Xie, Jiawen Zou, Junliang Lin, Kun Zhou, Xiangrong Lu, Molly M. Stevens

**Affiliations:** ^1^ Department of Materials, Department of Bioengineering and Institute of Biomedical Engineering, Imperial College London, London SW7 2AZ, UK.; ^2^ Department of Physiology, Anatomy and Genetics, Kavli Institute for Nanoscience Discovery, University of Oxford, South Parks Road, Oxford, OX1 3QU, UK.

## Abstract

3D soft bioscaffolds have great promise in tissue engineering, biohybrid robotics, and organ-on-a-chip engineering applications. Though emerging three-dimensional (3D) printing techniques offer versatility for assembling soft biomaterials, challenges persist in overcoming the deformation or collapse of delicate 3D structures during fabrication, especially for overhanging or thin features. This study introduces a magnet-assisted fabrication strategy that uses a magnetic field to trigger shape morphing and provide remote temporary support, enabling the straightforward creation of soft bioscaffolds with overhangs and thin-walled structures in 3D. We demonstrate the versatility and effectiveness of our strategy through the fabrication of bioscaffolds that replicate the complex 3D topology of branching vascular systems. Furthermore, we engineered hydrogel-based bioscaffolds to support biohybrid soft actuators capable of walking motion triggered by cardiomyocytes. This approach opens new possibilities for shaping hydrogel materials into complex 3D morphologies, which will further empower a broad range of biomedical applications.

## Teaser

Magnetically driven 2D-to-3D transformation strategy to engineer 3D soft bioscaffolds, empowering diverse biomedical applications.

## INTRODUCTION

Living creatures rely on a variety of three-dimensional (3D) soft components to perform activities. Learning from nature, researchers have been exploring approaches to engineer soft materials into 3D structures (*1*, *2*). This rapidly evolving field has attracted considerable attention and demonstrated immense potential in diverse areas such as soft robotics (*3*–*5*), soft electronics (*6*), tissue engineering (*7*, *8*), and organoid and organ-on-a-chip engineering (*9*, *10*).

3D printing has emerged as a widely used technique to create biomimetic 3D scaffolds due to its ability to precisely control the spatial organization of materials (*11*–*13*). During 3D printing, gravity aids with material deposition but also adds a constant downward force on the printed objects (*14*), which can cause deformation or collapse of the printed objects, especially if overhanging or thin features are present. To address these challenges, the supporting bath approach has been developed (*15*, *16*). In this innovative approach, a supporting bath physically supports the omnidirectional printing of soft materials, enabling the creation of intricate and physiologically relevant 3D structures (*17*–*19*). The success of this printing technique depends on the properties of the supporting bath. Specifically, the supporting bath material should exhibit Bingham plastic behavior: It should fluidize when subjected to stress from the moving nozzle, allowing for the extrusion of printed materials, and quickly return to a solid state when stress is removed, ensuring that the printed ink is firmly held within the bath. Moreover, the supporting bath should also assist in crosslinking to avoid ink diffusion. Thus, despite the numerous benefits offered by the supporting bath approach, its widespread application is now hindered by the limited availability of high-quality supporting materials and by the effort and expertise needed to optimize the slicing and printing parameters to match the properties of both the printing materials and the supporting materials (*20*).

Compared to delicate 3D structures, flat structures are much easier to directly fabricate into many desired shapes. Therefore, a 2D-to-3D transformation strategy would enable engineering of 3D structures from more accessible, lower-dimensional structures (*21*). Previous studies have shown that 2D-to-3D transformations can be triggered by spatially varying anisotropic swelling (*22*–*25*), compressive buckling (*26*, *27*), internal stress within materials (*28*, *29*), shape memory materials (*30*, *31*), and surface tension–driven floating (*32*), etc. These methods facilitate the manufacturing of intricate 3D geometries with reduced costs and time and subsequently led to the development of 4D printing techniques, in which materials adapt to changing environments (*22*, *33*, *34*). However, current strategies only offer shallow bending or rolling during shape morphing because they depend on the materials’ intrinsic properties to trigger the transformation and rely on specialized materials with responsive properties that are usually unsuitable for cell culture. Therefore, alternative methods are necessary to achieve large deformations, create complex structures at scale, and use diverse biocompatible materials.

**Fig. 1.**
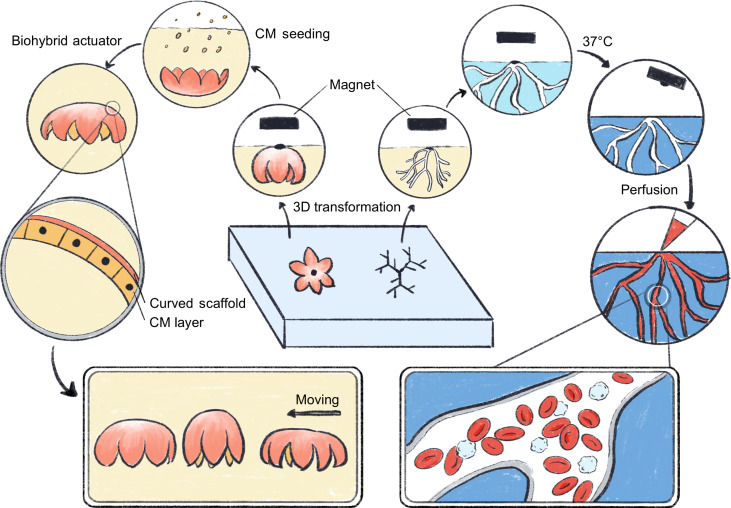
Conceptual illustration of magnetically driven transformation and their applications in engineering 3D perfusable vascular channels and a 3D biohybrid actuator with a walking motion. Figure credit: Y. Wang.

**Fig. 2.**
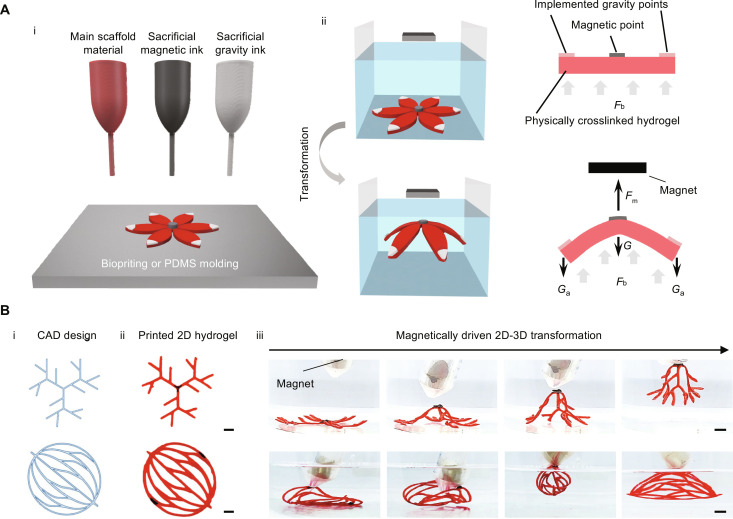
Concept of the fabrication strategy based on magnetically driven transformation. (**A**) Schematic illustration showing (i) deposition of materials into a flat hydrogel precursor, (ii) submerged transformation into a 3D morphology upon magnet application. *F*_m_ represents the magnetic force applied on the hydrogel, *F*_b_ represents the buoyancy force, *G* represents the gravitational force of the scaffold material, and *G*_a_ represents the gravitational force of the additionally implemented gravity ink. (**B**) 4D printing of 3D branching vascular geometries using the magnetically driven transformation strategy. The geometry designs were generated in AutoCAD (i), printed into flat hydrogel precursors (ii), and then transformed into 3D branching geometries using a magnet (iii). Scale bars, 1 cm.

In this study, we present a simple and versatile 3D biofabrication strategy that uses a remotely controlled magnetic force to transform 2D hydrogel precursors into complex 3D morphologies (Figs. 1 and 2A). The 3D morphology was supported by the combination of buoyancy, gravity, and magnetic forces after transformation and thus eliminated the need for physical supports. A biocompatible and thermo-responsive gelatin network was incorporated as a sacrificial support within the hydrogel to enable shaping of the structures before polymer crosslinking. Sacrificial magnetic ink and gravity ink were also used to assist in controlling the 2D-to-3D transformation. These sacrificial inks can be subsequently removed, leaving behind intact 3D bioscaffolds. This strategy allows for programmable shape morphing through adjustment of factors such as the initial 2D shapes, the temperature, or the spatial distribution of magnetic and gravity points. We used this shape-morphing strategy to engineer 3D bioscaffolds from multiple biocompatible hydrogels, including gelatin, methacrylated gelatin (GelMA), methacrylated hyaluronic acid (HAMA), methacrylated dextran (DexMA), and alginate. Furthermore, we demonstrated several potential biomedical applications of this manufacturing strategy. We fabricated 3D branching vascular channels in a crosslinkable bulk hydrogel and subsequently showed that these 3D channels are perfusable and can be endothelialized. We also generated curved thin-walled bioscaffolds for the fabrication of 3D soft actuators. Overall, the developed concept of using remote forces to support fragile 3D structures offers a unique shape-morphing and 3D fabrication strategy applicable to a wide range of material systems, thus opening up new possibilities for efficient 3D biofabrication to benefit researchers in biology, biomedical engineering, soft materials, and soft robotics.

## RESULTS

### Magnetically driven 3D transformation

A hydrogel scaffold transformation from flat to 3D was accomplished through remote control using magnetic force. The flat hydrogel precursor consists of three components: the main scaffold material, which will form the resulting structure, and sacrificial magnetic ink and gravity ink, which will aid the 3D transformation (Fig. 2A, i). All components contain physically crosslinkable gelatin, which can temporarily constrain the inks in place. The magnetic ink, composed of iron oxide particles and gelatin, was placed at the center of the flat hydrogel; while the gravity ink, composed of calcium carbonate and gelatin, was positioned at the extremities (Fig. 2A, i). Because of gelatin’s physically crosslinking property, these inks solidified quickly at the desired deposition points upon temperature decreases. To minimize damage to the delicate hydrogel scaffolds, the transformation was completed while the scaffold was immersed within a water bath and thus exposed to the buoyancy force, which partially counteracts the effects of gravity and prevents the entire structure from sagging excessively during shape morphing (Fig. 2A, ii). Application of an external magnetic field exposed the magnetic point to an upward magnetic force, lifting the hydrogel scaffold and initiated the 2D-to-3D transformation process with the assistance of a downward gravitational force (Fig. 2A, ii). In our strategy, the gravitational force is notably no longer a hindrance but a crucial element in 3D fabrication. The combination of buoyancy, gravity, and magnetic forces maintained the 3D structures until the materials were fully crosslinked and solidified, thus eliminating the need for physical supports from solid materials.

We investigated the feasibility of this approach to generate elaborate branched structures resembling 3D vascular networks. As 3D printing allows for flexible design and fast prototyping of scaffolds with complex features, we manufactured flat hydrogel precursors using an extrusion-based 3D printer (fig. S1). During printing, the print nozzle was heated to 45°C to ensure the smooth extrusion of the material, while the print bed was cooled to 4°C to ensure the rapid solidification of the material after extrusion (fig. S1B). The 2D hydrogel precursors were designed using CAD (Fig. 2B, i) and then printed using the extrusion-based 3D printer as shown in Fig. 2B, ii). Upon submersion into a water bath, the external magnetic field was applied to trigger 3D transformation (Fig. 2B, iii). The 3D networks were obtained within seconds and sustained in 3D for as long as the magnetic force was applied (movie S1). Generating these 3D branching structures through direct extrusion-based printing would be very difficult, as these delicate structures tend to collapse under their own weight. However, by using our magnetically driven transformation strategy, the printed flat hydrogels branches can easily be patterned into complex 3D shapes.

We also explored replica molding using poly(dimethyl)siloxane (PDMS) molds as an alternative technique to create the flat hydrogel precursors. For example, flat gelatin precursors were molded into various 2D geometries, including branching networks, spider shapes, star domes, and flowers (fig. S2) and easily transformed into 3D geometries using the magnet-triggered transformation strategy as shown in fig. S3. Thus, this magnetically driven transformation method overcomes some of the current limitations of molding and extrusion-based printing for creating delicate 3D structures while preserving flexibility and scalability.

### Multimodal control over the 3D transformation

**Fig. 3.**
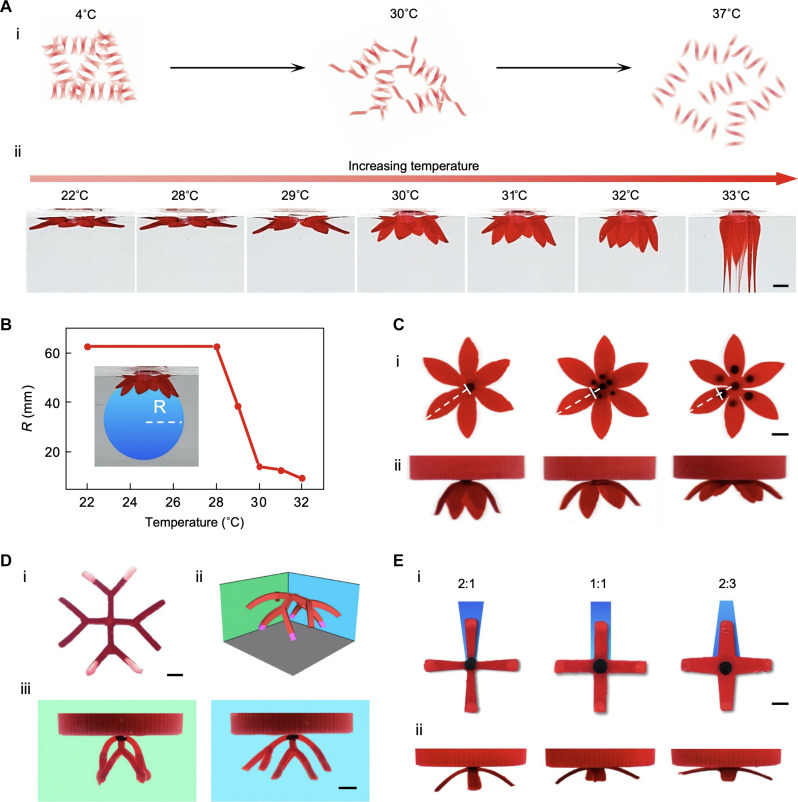
Multimodal control over the magnetically driven transformation. (**A**) (i) Scheme showing the thermal response of gelatin from the gel state to sol state. (ii) Transformation of a flower-shaped hydrogel precursor at different temperatures until it is melted at 33°C. (**B**) The radius of the curvature (R) when the flower-shaped hydrogel precursor was placed in water with increasing temperature. A blue circle was drawn under the image of the transformed hydrogel scaffolds to measure the radius. (**C**) Effect of magnetic center on 3D transformation. (i) Flat hydrogel precursors with increasing magnetic center area (the white dotted lines highlight the edge of the magnetic center in each hydrogel precursor and its distance from the center and the edge of the hydrogel precursor) and (ii) the corresponding 3D transformation of the precursors. (**D**) Effect of the gravity ink on 3D transformation. (i) Flat hydrogel precursors with two branches with the gravity ink (white tips) and two without. (ii) Schematic and (iii) experimental images of the transformed precursor with different levels of bending when viewed from the two different angles. (**E**) Effect of the 2D precursor structure designs. (i) Flat hydrogel precursors with different edge to center branch width ratio (2:1, 1:1, and 2:3) and corresponding 3D transformation; and (ii) corresponding 3D transformation of the precursors. Scale bars, 5 mm.

We next sought to explore the factors that can affect and control the transformation. The gelatin used to temporarily solidify the flat hydrogel precursor is a commonly used temperature-responsive material, which undergoes a gel-to-sol transformation as the temperature increases (Fig. 3A). To examine the impact of temperature on the transformation, we conducted tests using 20% (w/v) gelatin and used rheological measurements to evaluate the material’s response to temperature. The results indicated that the shear modulus of 20% (w/v) gelatin slightly decreases from 20° to 30°C but exhibits a substantial decrease beyond 30°C (fig. S3A). A temperature-sweep ranging from 22° to 33°C during the transformation of the hydrogel precursor confirmed that this decrease in shear modulus increases deformability (Fig. 3A, ii). The results demonstrated that the scaffold exhibits minimal deformation below 28°C and that the extent of transformation increases with temperature until the scaffold begins to melt at 33°C. In addition, measurements of the curvature radius revealed the relationship between the water bath temperature and the degree of achieved shape morphing (Fig. 3B). Specifically, the curvature radius of the transformed structure remained relatively constant as the temperature increased from 22° to 28°C but decreased substantially at temperatures above 28°C (Fig. 3B). We also examined the effect of temperature on the shape transformation of flat hydrogel precursors in various shapes. We found that a water bath temperature of 30°C compared to 25°C resulted in superior rendering of 3D structures for all tested shapes, when the amount of magnetic and gravity inks was held constant (fig. S3B). Thus, controlling the temperature allows us to tune the modulus of hydrogel precursors and subsequently the shape transformation.

The spatial distribution of magnetic ink and the volume of gravity ink, which are respectively responsible for the magnetic and gravity forces driving the transformation, can also be adjusted to provide shape morphing control. The position of the magnetic point can influence the morphology of the transformed structures as it determines the clamping point of the scaffold (Fig. 3C). When the distance of the magnetic point is closer to the extremity, the curvature radius of structure increases, suggesting a reduction in transformation. In addition, increasing the volume of the gravity ink resulted in a higher degree of shape morphing (Fig. 3D) by increasing the downward driving force provided by gravity. Furthermore, the original design of the 2D structure affects the degree of bending. We created structures with similar shapes but varying levels of connection at the bending point (Fig. 3E) and found that the structure with more connections at the bending point demonstrates weaker bending.

**Fig. 4.**
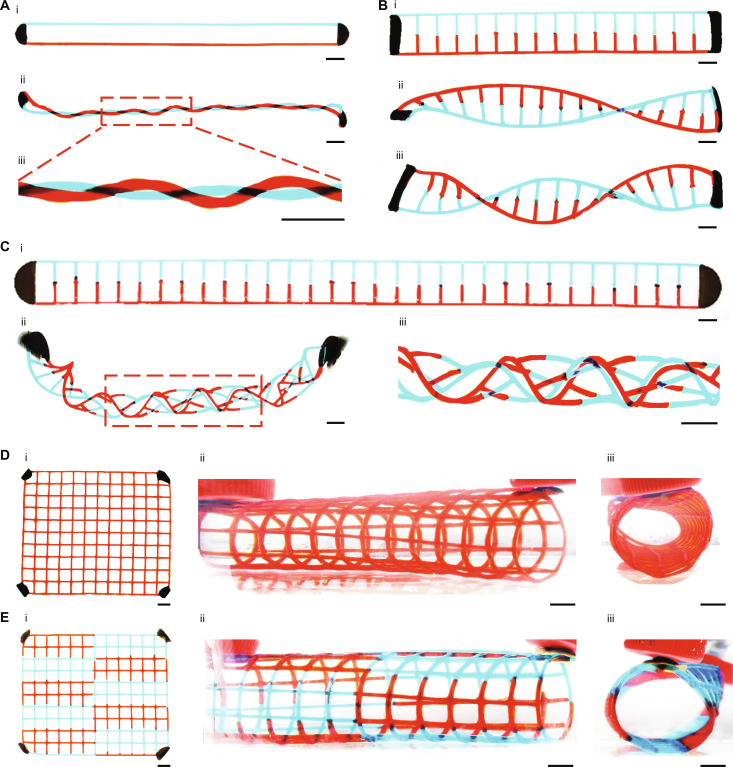
Patterning 3D structures using multimagnets. (**A**) Two strands (i) before and (ii and iii) after being twisted into a double intertwined helix with the assistance of two magnets. (**B**) DNA-like double helical structure (i) before and (ii and iii) after transformation. Transformed 3D double helical chains containing (ii) one knot and (iii) two knots. (**C**) Twisting of (i) an elongated ladder-like flat hydrogel precursor into (ii and iii) a braided tubular structure. (**D** and **E**) 3D tubular mesh consisting of (D) homogenous and (E) heterogeneous hydrogel mesh walls. (i) Flat hydrogel precursors containing four magnetic ink points. (ii and iii) Different views of the 3D tubular mesh. Scale bars, 1 cm.

Because the magnetic force triggers the morphing process, the implementation of more magnets is expected to introduce more complex magnetic forces, potentially triggering more versatile 3D transformations. As shown in Fig. 4A, we can easily weave two strands into an intertwined double helix by securing the two ends of a 2D hydrogel precursor using two magnets and then twisting the structure. In this structure, gelatin with red and blue food dye, used to represent two different materials, could easily be replaced by other materials to achieve multimaterial scaffolds. Similarly, a ladder-like 2D hydrogel precursor can transform into a DNA-like double helical structure (Fig. 4B). Further twisting of an elongated ladder-like flat hydrogel precursor can result in a woven tubular structure (Fig. 4C). In response to two magnets, a flat hydrogel mesh with four magnetic points at the four corners can be instantly rolled into a 3D tubular mesh (Fig. 4, D and E). These structures, particularly when they incorporate multimaterials in an interwoven structure, can be challenging to achieve using traditional fabrication strategies, which would necessitate frequent nozzle switching, potentially elevating the risk of printing failure. It is generally easier to fabricate structures with multiple materials in 2D than in 3D. Thus, our technique offers an easy and accessible way to produce complicated 3D structures composed of multimaterials.

Therefore, the shape morphing of the 2D hydrogel precursor into a 3D morphology can be finely tuned by adjusting the temperature, manipulating the opposing forces through controlled deposition of magnetic and gravity inks, and modifying the design of the original 2D structure. In addition, the magnetic points can serve as dynamic anchoring points, enabling intricate control over the transformation.

### Sacrificial templating of transformed scaffolds to create 3D branching channels

The development of 3D vascular branching networks could improve tissue engineering outcomes. Here, we explored the use of our magnetic transformation strategy to create bioscaffolds with 3D perfusable branching channels. For this purpose, we selected 20% (w/v) gelatin to fabricate 2D hydrogel precursors, which, after 3D transformation, can be sacrificed at cell-friendly temperatures to create channels. The magnetic ink, composed of iron oxide particles and 20% (w/v) gelatin, was placed onto the center of the 2D gelatin precursor. Then, the 2D gelatin precursor was placed into a crosslinkable hydrogel bath instead of a water bath.

**Fig. 5.**
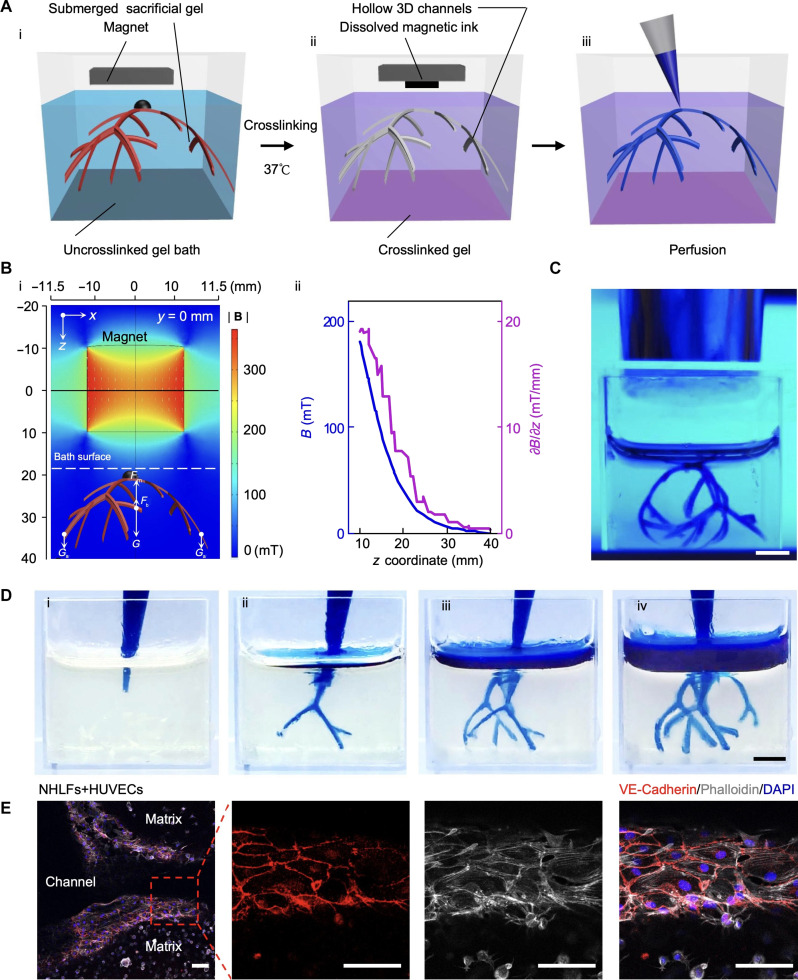
Magnetically driven transformation to create bioscaffolds with 3D branching vascular channels. (**A**) Scheme showing the steps to fabricate bioscaffolds with 3D branching vascular channels by magnetically driven transformation strategy. (i) Flat sacrificial gelatin precursor immersed in the uncrosslinked gel bath is transformed into 3D geometry when a magnet is applied. (ii) After crosslinking the gel bath under UV, the crosslinked scaffold is placed at 37°C with the magnet still placed on top to dissolve gelatin and remove the dissolved magnetic ink. (iii) Scheme showing the perfusion of the generated scaffold. (**B**) Magnetic field distribution analysis. (i) Finite element analysis of the magnetic field distribution caused by the applied magnet. (ii) Magnetic field distribution across the *z* coordinate. (**C**) UV crosslinking of the scaffold after the sacrificial precursor was transformed into a 3D morphology. Scale bars, 5 mm. (**D**) Scaffold perfusion with a blue dye demonstrated from (i) to (iv). Scale bars, 5 mm. (**E**) Immunostaining for VE-cadherin (Red), phalloidin (gray), and 4′,6-diamidino-2-phenylindole (DAPI) (blue) in the fibrin scaffold with HUVECs lining the channel wall and NHLFs within the fibrin matrix. Scale bars, 100 μm.

Here, we used GelMA prepolymer for the crosslinkable hydrogel bath as it can be readily crosslinked under ultraviolet (UV) and has biocompatibility suitable for tissue engineering applications. When a magnetic field was applied, the 2D gelatin precursor transformed into 3D morphology, indicated in Fig. 5A, i) as the submerged sacrificial gel. The GelMA prepolymer was then crosslinked under UV exposure, fixing the submerged sacrificial gel within the crosslinked GelMA. The submerged sacrificial gel made of 20% (w/v) gelatin was then sacrificed by incubation at 37°C (Fig. 5A, ii), leading to the generation of a perfusable scaffold (Fig. 5A, iii). We also simulated the magnetic field distribution (Fig. 5B, i). The magnet’s north and south poles were parallel to the *xz* plane, with the centerline directed toward the submerged sacrificial gel. The strength of the magnetic field on the *xz* plane decreased with increasing distance from the magnet (Fig. 5B). This observation suggests that the magnetic force exerted on the submerged sacrificial gel can be adjusted by altering the distance between the scaffold and the magnet. With the buoyancy force *F*_b_ and gravity force *G* and *G*_a_ remaining constant, the submerged sacrificial gel can float following adjustment of the applied magnetic force (*F*_m_). We confirmed experimentally that applying the magnet remotely results in 3D transformation as shown by a floating branching scaffold submerged in the GelMA bath and under UV exposure (Fig. 5C). However, it should be noted that producing a magnetic field for 3D transformation is not strictly confined to the use of a NdFeB magnet, which generates only gradient magnetic fields. For example, electromagnets arranged in either circular or saddle coil pairs could produce a spatially uniform magnetic field (*35*) to further enhance the controllability of the shaped structures. After UV crosslinking, the temperature was increased to 37°C to dissolve the submerged sacrificial gel made of 20% (w/v) gelatin, leaving behind hollow branching channels. The perfusion quality was assessed by injecting the channels with colored dye. As shown in Fig. 5D, we observed obvious penetration of the dye solution in each segment of the branches, indicating successful formation of channels. By changing the design of the flat hydrogel, hollow branching channels with different sizes and geometries were also achieved (fig. S4).

Then, we assessed the compatibility of this fabrication strategy with collagen and fibrin, two key protein-based natural hydrogels extensively used in tissue engineering. As shown in fig. S5A, collagen prepolymer was used as the bath material. The collagen bath undergoes a sol-gel transition at room temperature and thus becomes opaque over time. Following incubation at 37°C, the submerged sacrificial gel embedded in the collagen bath was removed, creating hollow channels. These hollow channels were perfused, as shown in fig. S5B. Using the same strategy, we also created 3D branching channels in fibrin and confirmed their perfusability (fig. S6).

Next, we tested if cells could survive the manufacturing processes. We used human umbilical vein endothelial cells (HUVECs), as they are commonly used for vascularization in vitro. We first assessed cell viability upon exposure to calcium carbonate and iron oxide particles. We used extreme conditions, exposing cells to doses of iron oxide particles as if they are cultured within magnetic ink or gravity ink at a density of 10 million/ml. Since both particles are removed after gelatin sacrifice, we selected a short-term exposure treatment of these particles (2-hour exposure) to model the effect of the manufacturing process (fig. S7A). Control groups included cells without exposure to these particles (no exposure) and cells exposed to these particles continuously for 7 days (long-term exposure). The results suggested that calcium carbonate particles do not decrease cell viability in either the 2-hour exposure or long-term exposure groups (fig. S7B). However, iron oxide particles significantly reduced cell viability for the long-term exposure group but not for the 2-hour exposure group (fig. S7C). Hence, the removal of iron oxide particles is crucial when applying this strategy for tissue engineering applications. To address this issue, the magnet was retained on top of the hydrogel scaffolds while we sacrificed the gelatin at 37°C (fig. S8A). As the gelatin dissolved, the iron oxide was released from the gelatin and attracted by the magnet (fig. S8B). This approach ensures that cells are not exposed to iron oxide particles for a substantial amount of time. To assess the potential impact of any residual particles on cell viability, we exposed cells to varying doses of iron oxide particles and monitored cell viability over a 7-day incubation period. The full dose (“1”) used in this test mimics the extreme condition, assuming that cells are cultured within the magnetic ink at a density of 10 million/ml. “1/2,” “1/5,” “1/10,” “1/20,” “1/50,” “1/100,” and “1/1000” are dilutions of the full dose. The results suggest that iron oxide particles do not have a significantly effect on cell viability if the dosage is reduced to less than 10% (1/10) of the full dose (fig. S9). Given (i) that cells are not directly cultured within the magnetic ink and (ii) that the magnetic ink constitutes only a small volume of the entire scaffold and can be easily removed by magnets after sacrifice, the utilization of magnetic particles does not have adverse effects on cells incorporated into the bioscaffolds.

To assess the ability of the 3D scaffolds with branching channels to promote cellular activity for tissue engineering applications, normal human lung fibroblasts (NHLFs) and HUVECs were cultured in the GelMA bioscaffolds. After 4 days of culture, both cell types exhibited good viability within the scaffold (figs. S10A and S11A). After 14 days of culture, cells surrounding the channels maintained good viability and established connections (figs. S10B; S11B; and S12, A and B). In contrast, cells located further away from the channels and within the gel displayed reduced activity over time and eventually underwent cell death (figs. S10B and S11B). These results indicate that the templated channels can be used to facilitate nutrient delivery and cell survival in the bioscaffold. Notably, the abundant presence of CD31, a marker for vessel formation, was observed around the hollow channels (fig. S12B). This observation confirms that the branching channels created by magnetically driven transformation can be endothelialized and used to engineer vascular structures.

We also assessed cell culture within the fibrin bioscaffolds. We incorporated endothelial cells in the sacrificial gel and fibroblasts in the fibrin bath. During the crosslinking process of the fibrin bath, endothelial cells remained within the sacrificial gel, and fibroblasts were within the fibrin matrix (fig. S13). After incubation at room temperature for 30 min, the fibrin was immersed in the culture medium and placed into the incubator. As the sacrificial gel dissolved, the endothelial cells attached to the fibrin scaffold walls, while the fibroblasts were embedded within the 3D fibrin matrix. After 7 days of incubation, we fixed and stained the fibrin scaffold with VE-cadherin and phalloidin. The staining further verified that the endothelial cells were growing within the channels, and the fibroblasts were distributed in the matrix (Fig. 5E). The clear VE-cadherin signal indicated the formation of tight junctions between the endothelial cells and the successful formation of the endothelial cell barrier.

### 3D transformed bioscaffolds can be crosslinked into freestanding structures

**Fig. 6.**
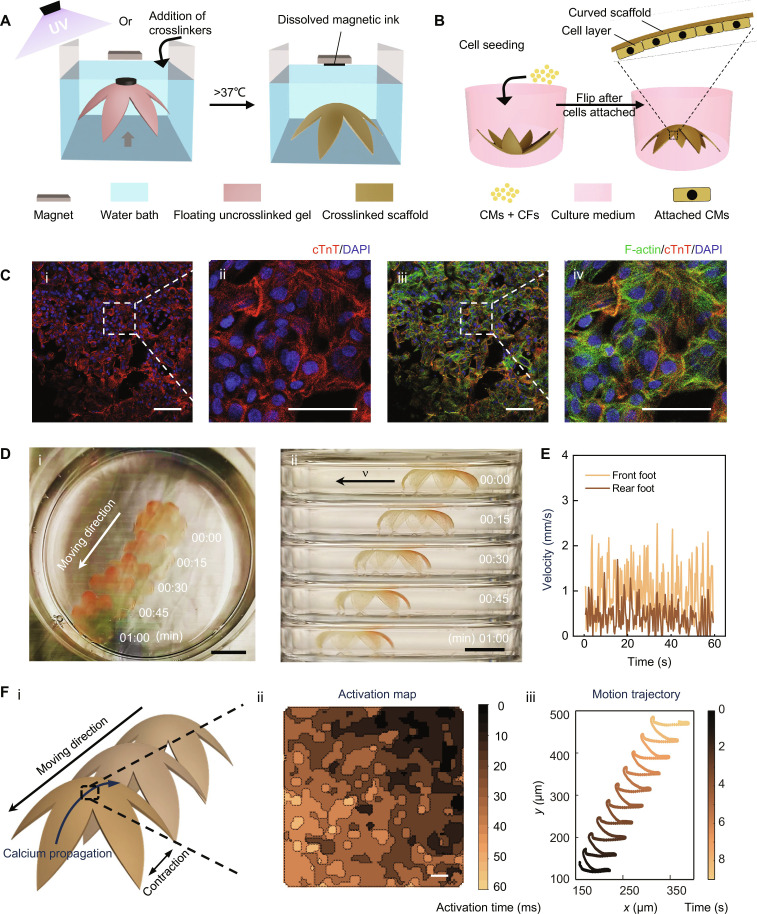
Magnetically driven transformation of 3D thin bioscaffolds enables the generation of hydrogel-based biohybrid actuator with a walking motion. (**A**) Scheme illustrates the process of creating 3D thin membrane-based bioscaffolds in a flower shape. Initially, a flat crosslinkable hydrogel precursor is transformed into a 3D morphology and then crosslinked when a magnet is placed above it. After incubation at 37°C, the crosslinked scaffold will be released from the magnet while gelatin dissolves, which simultaneously releases magnetic ink from the scaffold. (**B**) Scheme illustrating cell seeding and layer formation on the bioscaffolds. (**C**) Immunostaining of the cardiomyocytes cultured on the surface of the 3D thin bioscaffolds. cTnT (red), DAPI (blue), and F-actin (green). (i and iii) Low magnification. Scale bars, 50 μm. (ii and iv) High magnification. Scale bars, 25 μm. (**D**) (i) Top view and (ii) side view time-lapse photographs of walking biohybrid actuator (collected for 1 min). Scale bars, 1 cm. (**E**) Instant velocity of the front foot and rear foot over time. (**F**) (i) Schematic of the direction of the scaffold contraction, movement, and calcium propagation; (ii) calcium propagation mapping over time for the cardiomyocytes on the center of the scaffold (scale bars: 500 μm); and (iii) analysis of the motion of the scaffold.

Alternatively, we can use other crosslinkable hydrogel in combination with gelatin to create 3D-morphed structures. This approach allows for 3D transformed hydrogels to be crosslinked instead of sacrificed. The planar designs, whether molded or printed, can be transformed into 3D structures using the same magnetically triggered 2D-to-3D transformation in water as before. As depicted in Fig. 6A, once morphed into a 3D configuration, the structures can be crosslinked either by UV or by adding a crosslinker into the water bath, while the magnet stabilizes the structure.

After crosslinking, the 3D morphology is maintained upon removal of the magnet, resulting in free-standing 3D bioscaffolds (Fig. 6A and fig. S14). This shape morphing and crosslinking approach can be applied to various hydrogel systems, such as GelMA (fig. S14A), DexMA (fig. S14B), HAMA (fig. S14C), and alginate (fig. S14, D and E), all of which showed good 3D morphology after crosslinking. Moreover, we can assemble multilayer 3D-transformed scaffolds to create more intricate multilayer 3D features (fig. S14E).

However, when using UV to crosslink a material underwater, the crosslinking efficiency can be influenced by the depth of the material underwater due to variation in UV intensity. To investigate this dependence, we examined the Young’s modulus of GelMA crosslinked by UV for multiple durations in a 15-mm depth bath solution (deep bath) and a 5-mm depth bath solution, which only has a thin layer covering the materials (shallow bath). As shown in fig. S15, there was no significant difference between the deep and shallow bath groups at the same crosslinking durations. The Young’s modulus of groups crosslinked for 5 or 10 min was significantly lower than that of groups crosslinked for 15 or 20 min. Thus, the bath depth used in this study does not have a significant effect on the crosslinking efficiency; instead, crosslinking time is the dominant factor. However, no significant difference was observed between the 15- and 20-min groups, suggesting that GelMA is fully cured when exposed to UV for more than 15 min under the applied conditions. Thus, when a substantially deep bath is necessary for the creation of large scaffolds, the material can be crosslinked for a duration above the threshold to ensure a consistent material property throughout the scaffolds.

### Generation of a hydrogel-based biohybrid actuator with a walking motion

We next explored the functional utility of our 3D thin-walled bioscaffolds to form a biohybrid actuator. Biohybrid actuators which combine planar polymeric or hydrogel scaffolds and cardiac or muscle cells for locomotion have been extensively researched (*3*, *36*). These actuators successfully replicate the swimming motion of fish (*37*), ray (*38*, *39*) or jellyfish (*40*), and hold the potential to enable fluid pumping systems that mimic the function of the heart. Natural cardiac tissues have curvature that aids in pumping, but the planar scaffolds used in current biohybrid actuator systems do not include this feature. Moreover, while biohybrid actuators with walking motion powered by cardiomyocytes have been investigated (*41*, *42*), the use of biodegradable soft hydrogels has been rare, mainly due to the difficulty of engineering these materials into 3D thin-walled scaffolds. A recent study used stress-induced morphing to achieve a 3D printed cardiac patch with physiological curvature, although macroscopic contraction was not achieved due to the high mechanical resistance of the hydrogel scaffold (*43*).

To address this challenge, we sought to explore if the freestanding 3D thin-walled bioscaffolds generated by our strategy could facilitate the development of a biohybrid actuator with both desired curvature and macroscopic contraction and whether this design could result in a biohybrid robot capable of a walking motion. GelMA was chosen as the scaffold material due to its ability to incorporate the cardiomyocytes that have been used to trigger deformation of soft biohybrid actuators in previous literature (*38*). The flower structure was used because it can provide a large surface for cell attachment and also can stand freely under water to allow a potential walking motion.

To prepare the 3D biohybrid actuator, we initially prepared a flat hydrogel precursor composed of both gelatin and GelMA. After transforming the structure into a 3D configuration, the GelMA was UV-crosslinked. The thickness of the scaffold is minimized to achieve cell-triggered motion. The thickness of the scaffolds is approximately 300 μm (fig. S16A). Further reduction in thickness could pose challenges in material deployment, compromising the reproducibility of the scaffolds. After the fabrication, the scaffolds were incubated at 37°C overnight to remove gelatin, resulting in the final freestanding 3D thin-walled bioscaffolds (Fig. 6A). Both 5% (w/v) GelMA and 10% (w/v) GelMA were tested for scaffold fabrication, with the latter demonstrating better shape maintenance and reproducibility after gelatin removal (fig. S16B). Thus, we chose scaffolds made of 10% (w/v) GelMA for subsequent cell seeding. Human induced pluripotent stem cells (hiPSC) with GCaMP indicator-derived cardiomyocytes (GCaMP hiPSC-CMs), with more than 90% purity as tested by flow cytometry of cardiac troponin T (cTnT) staining (fig. S17), were then seeded onto the surface of the bioscaffold to generate the biohybrid 3D actuator (Fig. 6B). In addition, 10% cardiac fibroblasts were added to facilitate better cardiomyocyte attachment and functionality.

The cells on the scaffold were validated through staining of cTnT and F-actin (Fig. 6C), which revealed that the scaffold was well covered by cardiomyocytes with an isotropic cell growth and no alignment of cell bodies or sarcomeres. Even without guided cell alignment, the cell layer was strong enough to trigger a notable repeated contraction and relaxation of the entire scaffold due to the low bending resistance of the thin layer of hydrogel material (movie S2). To achieve a walking motion, we kept the bending side of the biohybrid 3D actuator facing down. The movement over time was recorded both from the top and from the side. From the recorded video (movies S2 and S3), we could observe the movement of the biohybrid 3D actuator triggered by the spontaneous beating of the cardiomyocytes. Images of the moving biohybrid 3D actuator over time are also shown in Fig. 6D. The different contraction force between the rear foot and the front foot, indicated by the different instant velocity of the two feet (Fig. 6E), was probably the major driving force to moving the scaffold forward. Calcium imaging of iPSC-CMs was performed to explore actuation at the cellular level. As the cardiomyocytes were derived from GCaMP iPSCs, calcium imaging could be done directly through fluorescent imaging. From the same calcium imaging video, we also tracked the movement of the scaffold. The results indicated that the direction of calcium propagation (Fig. 6F, ii) aligned with the movement of the flower-shaped soft actuator (Fig. 6F, iii), suggesting that contractions of iPSC-CMs contributed to the deformation of the soft actuator, generating rhythmic forward thrust that resembled a walking motion. Thus, using our magnetically triggered transformation strategy, we successfully engineered a 3D walking actuator solely composed of biocompatible hydrogels and cells. These advancements could potentially serve as an enabling platform for developing implantable engineered tissues and soft robots that interact with living systems.

## DISCUSSION

We have demonstrated a simple and versatile 3D biofabrication strategy that effectively transforms printed and molded flat soft hydrogel precursors into complex 3D structures. Unlike previously reported 2D-to-3D transformation strategies, our method does not rely on the material’s intrinsic properties. Instead, the responsive property comes from the sacrificial inks and the remote application of a magnetic field, making it compatible with a broader range of material systems and overcoming the requirement for precise predetermined material designs. Our strategy also offers easier control over 3D morphing via external forces and adjustment of shape morphing with temperature. Further, the remote magnetic force and gravity force applied onto the scaffolds eliminate the need for physical supports during fabrication, thus allowing easy fabrication of thin and soft bioscaffolds. In addition, these forces act in opposing directions, enabling substantial shape morphing and controlled bending without height limitations. This advantage allowed us to generate a branching 3D vascular structure, which was unachievable with previously reported 2D-to-3D transformation methods. Therefore, our strategy expands the possibilities of shape morphing in 3D biofabrication, both in terms of 3D structure and material diversity. Using this approach, we have achieved 3D branching networks and thin membrane arch structures composed of soft hydrogels. The resulting structures have been used as sacrificial templates to replicate the architectural patterns of branching vascular systems or as bioscaffolds that support manufacturing biohybrid soft actuators with a walking motion triggered by cardiomyocytes.

However, as the gelatin network is essential to help support the shape of the hydrogel precursor in our system, this strategy can currently only be applied to materials in an aqueous environment. To further expand the range of materials suitable for our 2D-to-3D transformation strategy, future studies can involve the design of alternative sacrificial materials that are compatible with oil-based polymers.

Overall, our strategy leverages remotely controlled forces to achieve precise control over material shape morphing and offers new opportunities for engineering complex 3D structures using soft biomaterials. This approach holds potential to facilitate future advances in engineered tissues, soft robotics, and 3D and 4D printing.

## MATERIALS AND METHODS

### Materials

All chemicals were purchased from Sigma-Aldrich unless otherwise specified. Materials used in cellular culture studies, such as photocrosslinkable polymers, were sterilized either through sterile filtration for solutions or via a 30-min UV treatment for powders.

The preparation of pork GelMA followed an established protocol (*44*). Methacrylic anhydride (0.6 g of methacrylic anhydride per 1 g of gelatin) was stirred dropwise into a 10% (w/v) gelatin solution (300 g of bloom, type A, from porcine skin) at 50°C. After 3 hours, the solution was centrifuged at 3500*g* for 3 min, and the supernatants were collected and diluted in four volumes of water. Subsequently, the solution was dialyzed (12- to 14-kDa molecular-weight cutoff) against 40°C water for 1 week, followed by a pH adjustment to 7.4 using 1 M NaHCO_3_.

For the preparation of fish gelatin methacryloyl (fish GelMA), 20 g of gelatin derived from fish skin was dissolved in 200 ml of carbonate buffer (pH 9.0 and 0.25 M concentration) by following an existing protocol (*45*). Subsequently, 1 ml of methacrylic anhydride was stirred dropwise into the fish gelatin under vigorous stirring for 5 hours at room temperature. The pH of the solution was adjusted to 7 using 1 M HCl, and then it was transferred to dialysis tubing (SpectraPor, standard RC 6- to 8-kDa molecular weight cutoff). The fish GelMA was dialyzed in the dark against water for 1 week.

HAMA was prepared following an established protocol (*46*). Methacrylic anhydride was added dropwise to a 1% (w/v) hyaluronic acid solution while maintaining a pH of approximately 8 over ice for 8 hours. The reaction continued overnight at 4°C, after which it was neutralized and dialyzed in water for 1 week.

DexMA was prepared following an existing protocol (*47*). Dextran (70 kDa; TCI Chemicals) was dissolved in 10% (w/v) LiCl/*N,N*′-dimethylformamide solution while stirring at 90°C. Once dissolved, the solution was cooled to 60°C and triethylamine was supplemented as a catalyst. Methacrylic anhydride was then stirred dropwise into the reaction at room temperature for 10 hours. The solution was subsequently dialyzed against water for 1 week.

### Cell culture

NHLFs (Lonza, CC-2512) and HUVECs (PromoCell, C-12203) were cultured using fibroblast growth medium-2 bullet kit (Lonza, CC-3132) and endothelial cell growth medium-2 (EGM-2) bullet kit (Lonza, CC-3162), respectively at 37°C and 5% CO_2_. Confluency was maintained around 50 to 90%. The medium was refreshed every 48 hours. Cells before passage 8 were used.

GCaMP human iPSC reporter cells were maintained in complete Essential 8 medium on Matrigel-coated plates. To prepare Matrigel-coated plates, Corning Matrigel human embryonic stem cell (hESC)–qualified Matrix (Corning, catalog #354277) was diluted in Dulbecco’s modified Eagle’s medium/F12 at 1:100 dilution ratio and then added into the six-well plates to incubate for 30 min. The cells were routinely passaged every 3 days with 1:12 split ratio. In addition, for the initial 24 hours after passaging, the cells were cultured with 10 μM Y-27632 (STEMCELL Technologies, #72307) to prevent apoptosis caused by cell dissociation.

### Cardiac differentiation

The process of cardiac differentiation was optimized on the basis of a previously reported protocol (*48*). When the cells reached approximately 85% confluence, the medium was switched to differentiation medium, which consisted of RPMI (Gibco, 11875-093) supplemented with 2% (v/v) B27-insulin supplement (Gibco, 17504-044). From day 0 to day 2, the differentiation medium with 6 μM CHIR99021 (Tebu-bio, 04-0004-02) was added and replaced with a fresh differentiation medium on day 2. The differentiation medium with 2.5 μM Wnt-C59 (Stratech, S7037-SEL) was used from day 3 to day 5 and was replaced with a fresh differentiation medium on day 5. On day 7, the medium was switched to RPMI supplemented with 2% (v/v) B27 supplement and refreshed every other day. Starting from day 7, spontaneous contraction of the cells was observed. For the metabolic selection of cardiomyocytes, the medium was changed every other day to RPMI without glucose (Gibco, 11879-020) supplemented with 2% (v/v) B27 supplement and 5 mM sodium lactate (Sigama-Aldrich, L4263) from day 11 to day 17. After metabolic selection, the differentiated cardiomyocytes were dissociated using collagenase type II (Thermo Fisher Scientific, 17101-015) for 3 hours and replated onto a Matrigel-coated plate for further experiments. The cardiomyocytes were maintained in a maintenance medium consisting of RPMI supplemented with 2% (v/v) B27 supplement.

The purity of cardiomyocytes was evaluated by flow cytometry. First, detached hiPSCs and CMs were fixed with 4% (w/v) paraformaldehyde (PFA) (Electron Microscopy Sciences, #15710) for 10 min, followed by permeabilization using 0.1% (v/v) Triton X-100 (Sigma-Aldrich, X-100) for 10 min. Subsequently, the cells were stained with Alexa Fluor 647 isotype antibody (BD Bioscience, 557732) as control and Alexa Fluor 647 mouse anti-cTnT (BD Bioscience, 565744), which was diluted 1:400 in a 1% (v/v) fetal bovine serum (FBS) solution in Dulbecco’s phosphate-buffered saline (DPBS) for overnight at 4°C. Cells were washed with DPBS with 1% (v/v) FBS between each step. Last, the stained cells were analyzed using flow cytometry.

### Preparation of magnetic and gravity inks

To prepare magnetic and gravity ink, 750 mg of iron(II,III) oxide powder (Nanoamor) and 500 mg of calcium carbonate (Sigma-Aldrich) were added in 5 ml of 20% (w/v) gelatin solution, respectively. Allura Red (1 mg/ml; Sigma-Aldrich) was incorporated into the gelatin solution for better visualization. For usage, all inks were kept in a 60°C oven to ensure their liquid state. For storage, the inks were kept in the fridge.

### Rheological measurements

A rheometer (MR 302, Anton Paar) was used to conduct theological measurements. A 20% gelatin heated to 60°C was loaded for the tests. Oscillatory strain sweeps (10^−1^ to 10^3^%) were conducted at 22°C using a frequency of 1.5 Hz. Oscillatory frequency sweeps (0.1 to 100 Hz) were performed at 22°C using a shear strain of 1%. To measure the thermal responsivity of the hydrogels, we conducted oscillatory temperature sweeps using a temperature-controlled plate. The temperature was reduced to 4°C and then increased to 37°C. A frequency of 1.5 Hz and a strain of 1% were maintained throughout these experiments.

### Fabrication of flat hydrogel scaffolds

Flat hydrogel scaffolds with customized shapes were fabricated using either a PDMS molding process or a bioprinting technique. For the PDMS molding process, positive molds were initially printed using a 3D printer (Original Prusa SL1S SPEED) with a commercial ink (Vibrant Orange Resin Tough). Subsequently, the printed molds underwent a 30-min soaking in isopropanol under ultrasound treatment, followed by drying in a 65°C oven for 2 hours. The surfaces of the printed structures were then vapor-coated with trichloro(1*H*,1*H*,2*H*,2*H*-perfluorooctyl) silane for 4 hours after O_2_ plasma activation (100 W at 1 mBar O_2_ for 5 min). The PDMS was mixed at the ratio of 10:1 polymer to crosslinker, degassed, added into the positive molds, and placed in an oven at 85°C for 2.5 hours. Subsequently, the PDMS negative molds were peeled off from the solid templates. Positive gelatin molds were then created by adding 20% (w/v) gelatin solution into the PDMS molds after coating them with a 40% (w/v) Pluronic F-127 solution (P2443). Specifically, molds were filled with the prepared 20% (w/v) gelatin solution. During the filling process, the molds were placed on a heat pad at 60°C to prevent the gelation of gelatin solution. Once filled, the molds were cooled to 4°C for 5 min in a humid environment. Then, magnetic and gravity inks were placed in the center and branch tips of the solidified gelatin, followed by another 5-min incubation at 4°C in a humid environment. The resulting flat hydrogel precursors were spontaneously released when placed in water.

Flat hydrogel scaffolds can also be fabricated using a 3D bioprinter (SunP BioMaker 2). A 20% (w/v) gelatin precursor solution was loaded in a 5-ml pipette with a metal tip and introduced in the heated printer nozzle set at a temperature of 45°C. The gelatin bioink was allowed to equilibrate before printing on a precooled printer bed (4°C) coated with a thin layer of 40% (w/v) Pluronic F-127 solution. A layer height of 0.3 mm, a printing speed of 12 mm/s, and an extrusion speed of 1.6 mm^3^/s were found to be optimal. After printing, the magnetic ink and gravity ink were added to the desired segments of printed figures, allowing the inks to integrate for 5 min over the 4°C printing bed.

### Magnetically driven 2D-to-3D transformation of scaffolds

The flat hydrogel precursors were transformed within a water bath without the requirement of any additional supporting material. A cylindrical NdFeB magnet (diameter and height of 20 mm) was used to apply an external magnetic field to achieve the 2D-to-3D transformation of the hydrogel scaffolds. To induce transformation, the magnet was positioned above the water level with a plastic interface, which prevented direct contact between the hydrogels and the magnet and helped to keep the magnet in place. To maintain the transformed shape over time, the magnet was left in place on top of the interface.

For the transformation using multiple magnets, the magnets were first placed close to the designated magnetic points. This will help the desired points be selectively grasped. Then, the twisting or the lifting of the scaffolds can be controlled by handling the magnets.

The magnetic field and field gradient distribution generated by the cylindrical NdFeB magnet (First4Magnets) was simulated using a commercial finite element analysis software (COMSOL Multiphysics 5.4). The remanent magnetization of the magnet was 4.45 × 10^5^ A/m.

### Measurement of the curvature radius

The images and videos of the transformed structure were processed using either Microsoft PowerPoint or Adobe Photoshop. A circle matching the curvature of the transformed structure was added. The radius of this circle was then measured and compared to the scale bar to determine the radius of the structure’s curvature.

### Fabrication of bioscaffolds with 3D branching vascular channels

Sterile gelatin powder was dissolved in sterile PBS to prepare 20% (w/v) gelatin. PDMS molding process was used to prepare the flat gelatin templates. The templates were then transferred to a tank filled with sterile PBS. Subsequently, HUVECs at a concentration of 8.0 × 10^6^ cells/ml, were mixed with a GelMA prepolymer solution. The GelMA prepolymer solution contains 10% (w/v) fish GelMA and 5 mM lithium phenyl-2,4,6-trimethylbenzoylphosphinate (LAP) in PBS. The PBS in the tank was replaced by the GelMA prepolymer solution containing HUVECs. Through magnetically driven transformation, a 3D sacrificial gelatin structure with branches was achieved within the GelMA prepolymer solution and maintained over time by leaving the magnet in place. UV exposure was conducted from the bottom and top of the tank, respectively, for 5 min to crosslink the GelMA solution and yield the GelMA scaffold with HUVECs. To prevent oxygen inhibition, an additional 2-min UV light treatment was performed after adding 2 mM LAP solution to cover the scaffold. The entire scaffold was then incubated in EGM-2 culture medium and transferred to a 37°C incubator to remove the gelatin templates. A magnet was continuously applied on top of the well plate for 30 min to ensure complete removal of the magnetic ink. Last, the scaffolds were removed from the tank and cultured on a shaker in the incubator. Cells at a concentration of 8 × 10^6^ cells/ml were also used to evaluate the effect of the 3D branching vascular channels on cellular activity.

To prepare perfusable fibrin scaffolds, we used PDMS molding to prepare the flat gelatin templates. The templates were then transferred to a tank filled with PBS. Subsequently, the PBS was removed. Thrombin (6 U/ml; T7099) and fibrinogen (30 mg/ml; F8630) were added at 1:1 ratio into the tank. The magnet was then placed on top of the scaffold to trigger 3D transformation. After incubating the materials at room temperature for 30 min, the fibrinogen was crosslinked, and the fibrin scaffold was placed into 37°C incubator overnight for the removal of gelatin. For cell culture within the fibrin scaffolds, HUVECs (10 × 10^6^ cells/ml) were included into gelatin templates and NHLFs (5 × 10^6^ cells/ml) were mixed within the fibrinogen solution. HUVECs were colored by Green CMFDA Dye (Thermo Fisher Scientific, C7025), and NHLFs were colored by Orange CMRA Dye (Thermo Fisher Scientific, C34551) for visualization under a microscope. Fibrin scaffolds containing HUVECs and NHLFs were cultured for 7 days before being fixed by 4% PFA (w/v).

To fabricate perfusable collagen scaffolds, the prepolymer solution added into the gel is the mixture of 10× PBS, 7.5% NaHCO_3_ (v/v), 1× PBS, and collagen (5 mg/ml) for achieving collagen (2.5 mg/ml) with pH 7.4. The collagen scaffolds were crosslinked at room temperature for 50 min before being placed in the incubator at 37°C. To visualize the 3D branching vascular channels and assess the extent of perfusion within the bulk hydrogel, blue food dye was injected into the lumen of the topmost channel opening and allowed to flow throughout the entire network.

### LIVE/DEAD staining

The cell-laden hydrogels were washed three times with DPBS buffer. Subsequently, cells were immersed in 1 ml of DPBS solution containing Calcein AM Viability Dye (0.5 μl/ml) for live cell staining (Thermo Fisher Scientific, C0875) and ethidium homodimer-1 (2 μl/ml; Thermo Fisher Scientific, E1169) for dead cell staining for 30 min, followed by gentle DPBS buffer washing. All microscopic images were captured under Zeiss Axio Observer (Carl Zeiss, Oberkochen, Germany).

### Cell viability test

HUVECs were seeded at 20,000 per well in 96-well plates. Using the fabrication method, the highest concentration of calcium carbonate in the gel is 100 mg/ml, and the highest concentration of iron oxide is 150 mg/ml. Assuming a cell seeding density in the gel of 10 million cells/ml, we added 0.9 mg of calcium carbonate or 1.35 mg of iron oxide to each well to simulate the highest exposure of cells to these particles. The effect of iron oxide and calcium carbonate on cell viability was evaluated for short-term (2 hours) and long-term (7 days) treatment and tested over time using an alamarBlue assay (Thermo Fisher Scientific).

To test the effect of iron oxide concentration, varying dosages of iron oxide particles were sequentially diluted from the highest dosage of 1.35 mg per well, into 1/2, 1/5, 1/10, 1/20, 1/50, 1/100, and 1/1000. Cell viability was tested using an alamarBlue assay at day 3, day 5, and day 7.

### Crosslinking the 3D transformed scaffolds

To enable permanent crosslinking of the transformed 3D structure, 15% (w/v) gelatin, for temporary shaping of the structure, was mixed with chemically crosslinkable hydrogels. 3D transformed patterns made from 10% (w/v) pork GelMA, 5% (w/v) HAMA, and 10% (w/v) DexMA hydrogel were crosslinked within PBS containing 2 mM LAP under UV for 10 to 20 min. In contrast to UV crosslinkable materials, alginate patterns were first 3D transformed in a water bath; then, 500 mM calcium chloride solution was added into the water bath until a final concentration of 50 mM calcium chloride was reached. Appropriate crosslinking of these patterns was assessed by removing the magnet and visually confirming their ability to maintain 3D integrity in the absence of any magnetic force.

### Compression tests

Prepolymer containing 15% (w/v) gelatin, 10% (w/v) pork GelMA, and 2 mM LAP was used to prepare the scaffolds for the compression tests. Syringes (1 ml; BD, Luer slip syringe) with the tip being cut were used as molds for the fabrication. Prepolymers (110 μl) were added to the syringe. After being cooled down in the fridge for 5 min, the polymers were physically crosslinked to form cylinders with a diameter of 4.72 mm and a height of 6.29 mm. Then, the cylinders were placed in a tank containing 2 mM LAP solution for UV crosslinking. The depth of the LAP solution within the tank was 15 mm (deep bath) or 5 mm (shallow bath). In the shallow bath, the materials are only covered by a thin layer of bath solution. The crosslinking time was 5, 10, 15, or 20 min. At least five scaffolds were prepared for each group. The scaffolds were placed in PBS and incubated at 37°C overnight before the compression tests. Compression tests were conducted on a uniaxial mechanical tester (EletroForce 3200, TA Instruments) with a 2 N load cell. Ramp compression was applied at a speed of 0.05 mm/s. The Young’s modulus was determined as the slope of the linear line between 2 and 6% strain of the stress-strain curve.

### Generation of hydrogel-based biohybrid actuator

Porcine GelMA was chosen as the scaffold material for preparing the biohybrid actuator due to its advantage in allowing cell attachment. Initially, a prepolymer solution containing 15% (w/v) gelatin, 10% (w/v) pork GelMA, and 2 mM LAP in PBS was prepared. This prepolymer solution was used to create a flat hydrogel precursor using the flower molds and the PDMS molding method. The flat hydrogel precursor was then transferred to a water bath containing PBS with 2 mM LAP. Upon magnet application, the flat hydrogel precursor was converted into a 3D structure, followed by UV treatment for 15 min. Subsequently, the 3D structure was heated to 37°C to liquefy the gelatin within the network. The 3D-shaped scaffold was washed three times with PBS before being immersed in a cardiomyocyte culture medium and incubated overnight. The cardiomyocyte culture medium was composed of RPMI medium supplemented with 2% (v/v) B27 supplement and 10% (v/v) FBS. The scaffold was then transferred to a 12-well plate treated with anti-adherence rinsing solution (STEMCELL Technologies) and with 2 ml of cardiomyocyte culture medium. A total of 3.3 million cells (3 million GCaMP hiPSCs-derived cardiomyocytes and 0.3 million human cardiac fibroblasts (PromoCell, C-12375) were seeded onto each 3D scaffold by directly adding the cell suspension to the well plate of each scaffold. The culture medium was replaced twice a week.

### Motion analysis

The motion of the hydrogel-based biohybrid actuators was captured and recorded using a digital camera. The recorded videos were subsequently analyzed using an open-source software (Tracker, version 6.1.3; https://physlets.org/tracker/). To determine the position of the biohybrid actuators, they were tracked manually by analyzing the frames of the videos, which enabled the collection of a series of coordinates of the biohybrid actuators. By processing all frames of the recorded videos, the obtained coordinates were used to calculate the displacement and speed of the biohybrid actuators.

### Calcium imaging and propagation mapping

For calcium imaging, the cell culture medium was replaced with Tyrode’s solution (Hepes-buffered, Thermo Fisher Scientific). The samples were placed in the live-cell chamber of Zeiss Axio Observer kept at 37°C with 5% CO_2_ and humid environment. Alexa Fluor 488 channel was used for imaging, with an exposure time of 10 ms and an imaging duration of 15 s.

Images were processed using ImageJ software (National Institutes of Health, Bethesda, MD). To analyze the calcium propagation, the frames within the video were processed in ImageJ and made binary to highlight the brightest region in each frame. Then, the binary images were colorized in the MATLAB to show the propagation profile. The TrackMate function in ImageJ was applied to track the movement of the scaffold.

### Immunostaining and imaging

A 4% (w/v) PFA was used to fix the samples at 4°C overnight, followed by PBS washing three times, and then kept in the fridge for further staining. To stain HUVECs, the samples were permeabilized by the permeation solution containing 5% (w/v) bovine serum albumin (BSA) solution (Sigma-Aldrich, A2153) and 0.5% (v/v) Triton X-100 and further blocked by 5% (w/v) BSA in the fridge overnight. Then, samples were incubated in 1% (w/v) BSA solution containing CD31 antibody (Thermo Fisher Scientific, MA5-29475) or VE-cadherin antibody (Thermo Fisher Scientific, 14-1449-82) with 1:100 dilution at 4°C overnight. After washing three times using PBS, the samples were incubated at 4°C overnight in 1% BSA (w/v) solution containing a secondary antibody (Thermo Fisher Scientific, A31573 for CD31 and A21203 for VE-cadherin) with 1:300 dilution. The samples were then incubated with 4′,6-diamidino-2-phenylindole (DAPI) (Thermo Fisher Scientific, D1306) at 1:1000 dilution for 5 min and washed three times with PBS. To characterize NHLFs, the samples were permeabilized by the permeation solution containing 5% (w/v) BSA and 0.6% (v/v) Triton X-100 and further blocked by 5% (w/v) BSA in the fridge overnight. Then, scaffolds were incubated in Alexa Fluor 488 phalloidin (Thermo Fisher Scientific, A12379) solution (1:500 dilution) at room temperature for 1 hour. After DAPI staining, the samples were washed three times with PBS.

To stain cardiomyocytes, the samples were permeabilized with a permeation solution containing 5% (w/v) BSA and 0.2% (v/v) Triton X-100 and further blocked by 5% BSA at room temperature for 2 hours. Then, scaffolds were incubated in 1% (w/v) BSA containing Alexa Fluor 647 mouse anti-cTnT (BD Bioscience, 565744) at 4°C overnight. Subsequently, Alexa Fluor 488 phalloidin was diluted at 1:500 dilution ratio and incubated with the scaffolds at room temperature for 1 hour. After DAPI staining, the samples were washed three times with PBS.

The images were captured using a Leica SP8 confocal microscope and processed using the ImageJ software.
