# Supplementary material for: Magnetically driven formation of 3D freestanding soft bioscaffolds
Source: Sci Adv. 2024 Feb 2;10(5):eadl1549. doi: 10.1126/sciadv.adl1549 (PMC10836728; doi:10.1126/sciadv.adl1549)
Supplement: Supplementary file 1 — Figs. S1 to S17 Legends for movies S1 to S3 [file sciadv.adl1549_sm.pdf]

Science Advances
AAAS

Supplementary Materials for

Magnetically driven formation of 3D freestanding soft bioscaffolds

Ruoxiao Xie *et al.*

Corresponding author: Molly M. Stevens, molly.stevens@dpag.ox.ac.uk

Sci. Adv. **10**, eadl1549 (2024)
DOI: 10.1126/sciadv.adl1549

The PDF file includes:

Figs. S1 to S17
Legends for movies S1 to S3

Other Supplementary Material for this manuscript includes the following:

Movies S1 to S3

Supplementary Figures

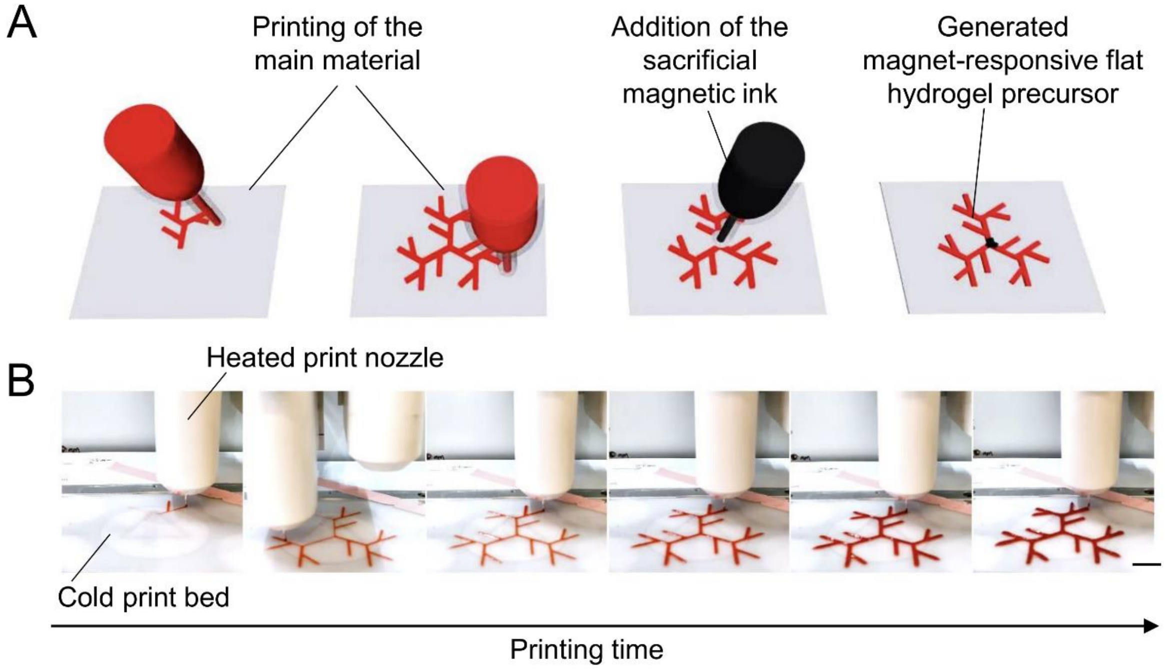

Fig. S1. Printing the flat hydrogel precursors. (A) Schematics and **(B)** photos showing the printing of hydrogel inks into flat hydrogel precursors. (Scale bars: 1 cm).

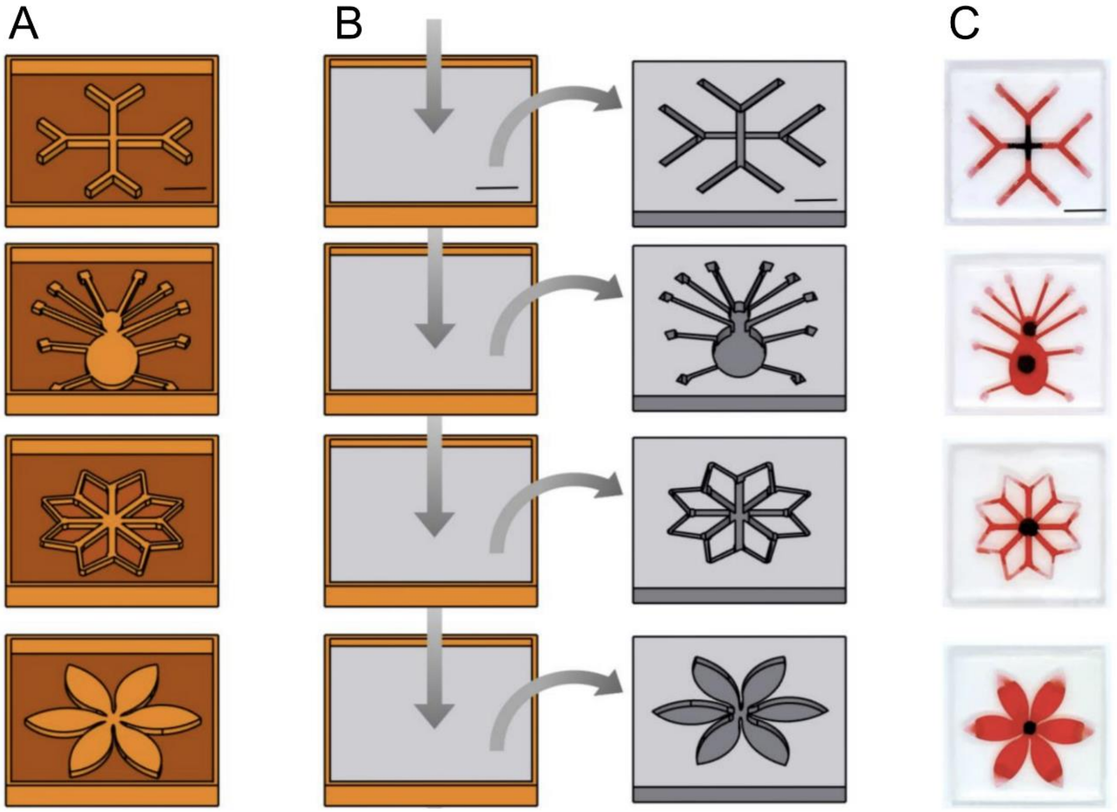

Fig. S2. Molding the flat hydrogel precursors. (A) Schematics showing the printed molds, **(B)** PDMS molds casting, and **(C)** the PDMS molds loaded with hydrogel inks for making molded flat hydrogel precursors. (Scale bars: 1 cm).

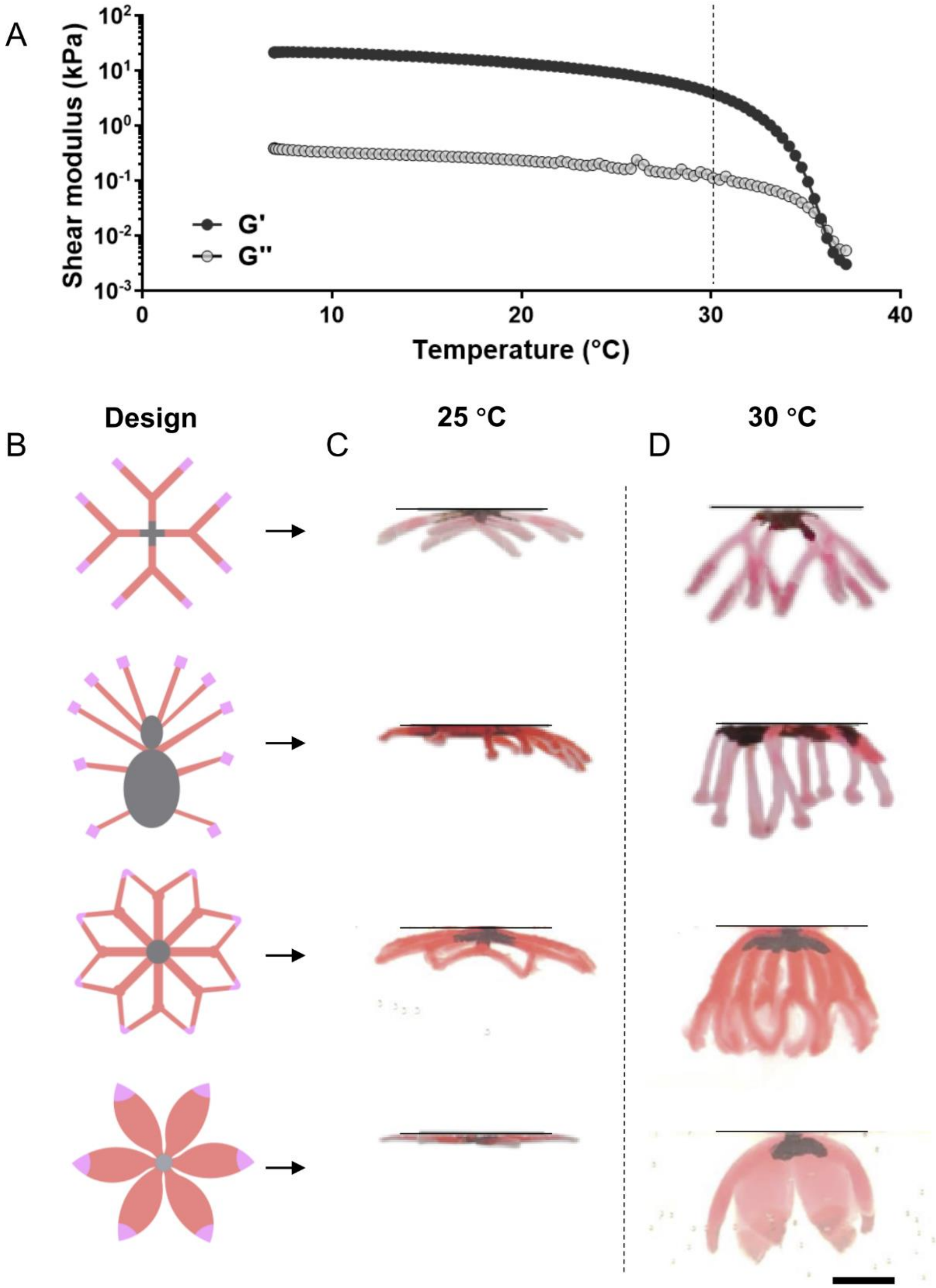

Fig. S3. The effect of temperature on the material property and the 3D transformation of the different patterns. (A) Storage (G′) and loss (G″) moduli of 20% (w/v) gelatin during temperature sweep (from 7 °C to 37 °C). **(B)** Schematics of the molded flat hydrogel precursors, and the photos of their 3D transformation when at **(C)** 25 °C and **(D)** 30 °C. (Scale bars: 1 cm).

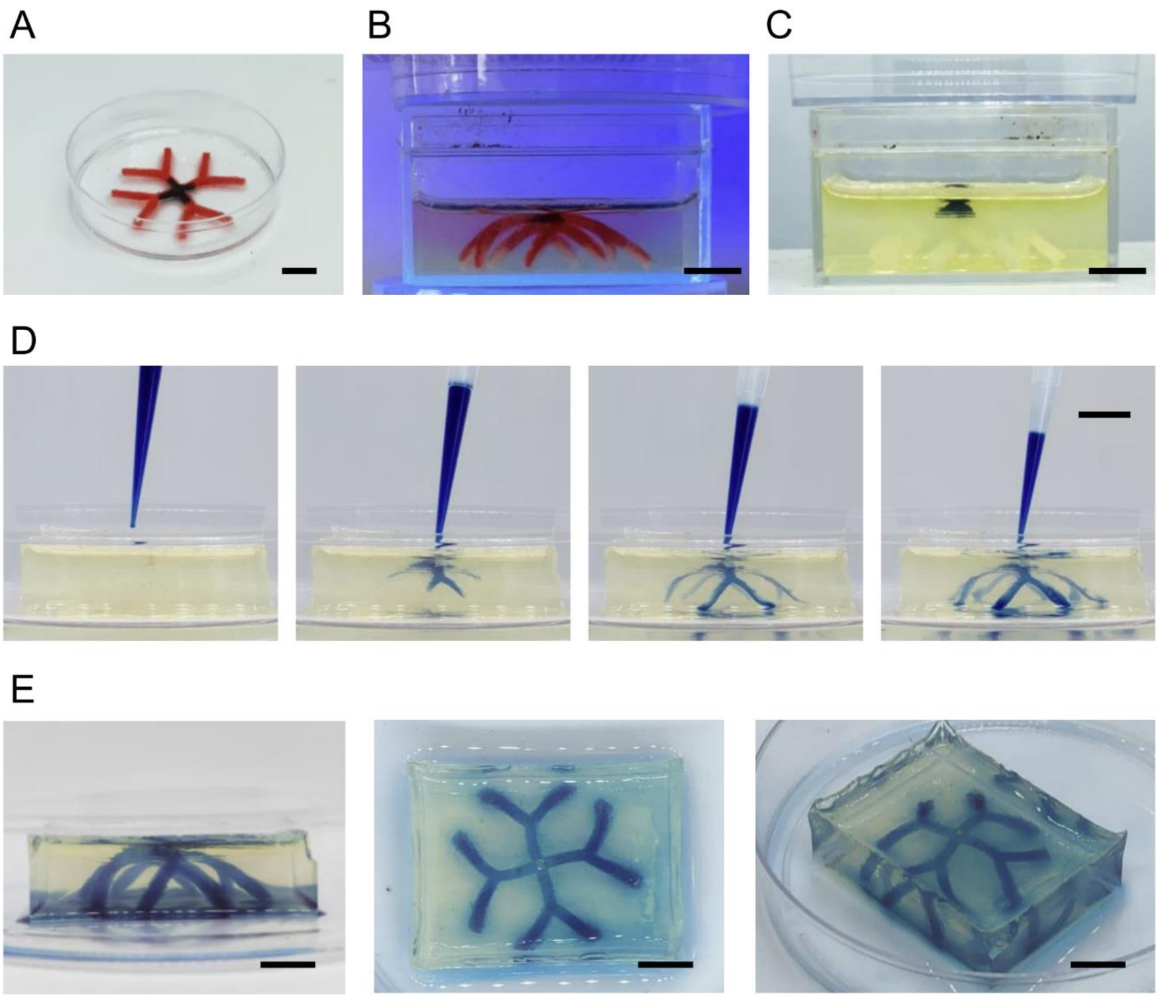

Fig. S4. Fabrication of GelMA scaffolds with 3D branching vascular channels by magnetically driven transformation. (A) Molded flat sacrificial hydrogel precursors; **(B)** magnetically driven transformation of the sacrificial hydrogel precursor into 3D morphology followed by UV crosslinking of the GelMA bath; **(C)** crosslinked bath material (GelMA) with embedded sacrificial 3D branching hydrogels, which can be sacrificed when placed into 37° C incubator to generate bioscaffolds with 3D branching vascular channels; **(D)** perfusion of blue dye solution into the 3D branching vascular channels; **(E)** different views of a perfused bioscaffold with 3D branching vascular channels. (Scale bars: 10 cm).

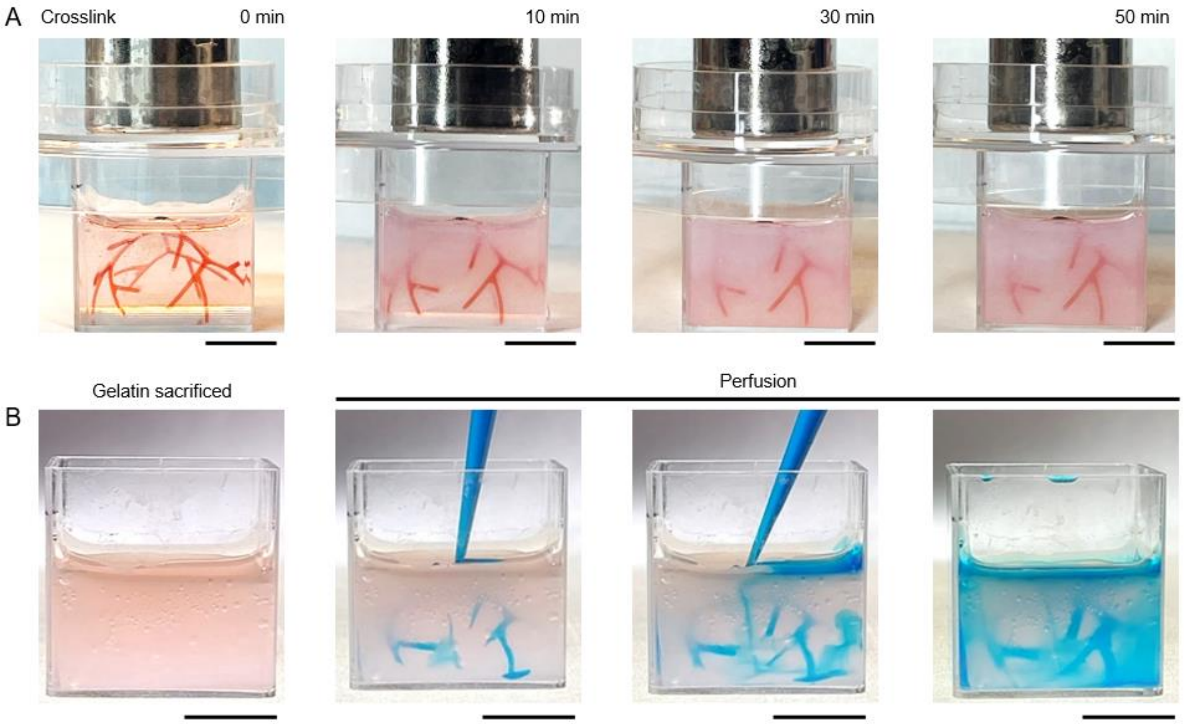

Fig. S5. Fabrication of collagen bioscaffolds with 3D branching channels by magnetically driven transformation. (A) Time-lapse images showing the crosslinking process of the collagen bath at room temperature. **(B)** Perfusion of blue dye solution into the 3D branching channels within crosslinked collagen after gelatin removal. (Scale bars: 1 cm).

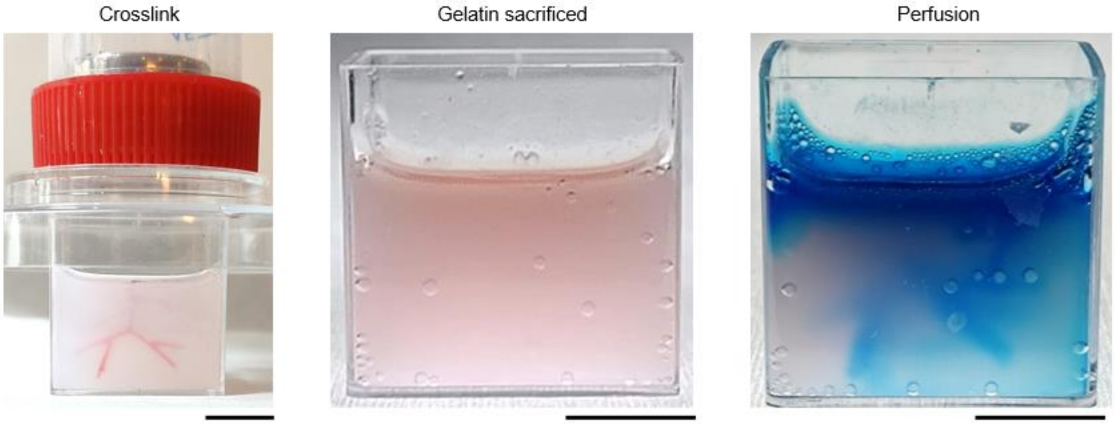

Fig. S6. Fabrication and perfusion of fibrin bioscaffolds with 3D branching channels. (Scale bars: 1 cm).

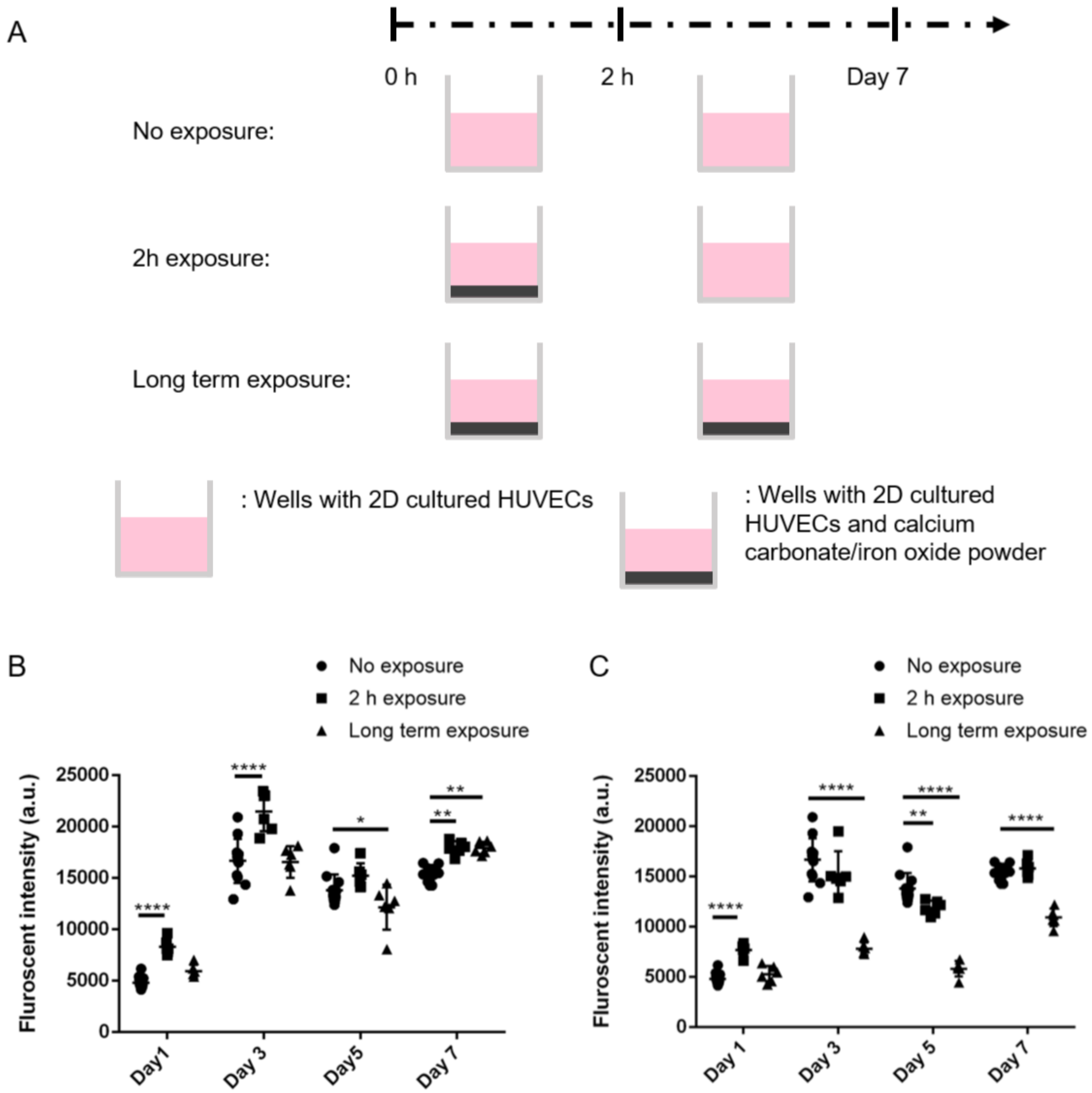

Fig. S7. Effect of $CaCO_3$ and iron oxide powders on cell viability tested using Human Umbilical Vein Endothelial Cells (HUVECs). (A) Scheme showing the three groups being tested for each powder. “no exposure” is a control group, “2 h exposure” is the group with powders kept with cells for 2 hours and then removed, and “Long term exposure” is the group with powders kept with the cells for 7 days. **(B)** The effect of $CaCO_3$ powders on HUVECs viability. **(C)** The effect of iron oxide powders on HUVECs viability. (Data are shown as average ± s.d., $n = 6$ from one experiment. *$p < 0.05$, **$p < 0.01$, ***$p < 0.001$, ****$p < 0.0001$ based on two-way ANOVA test.).

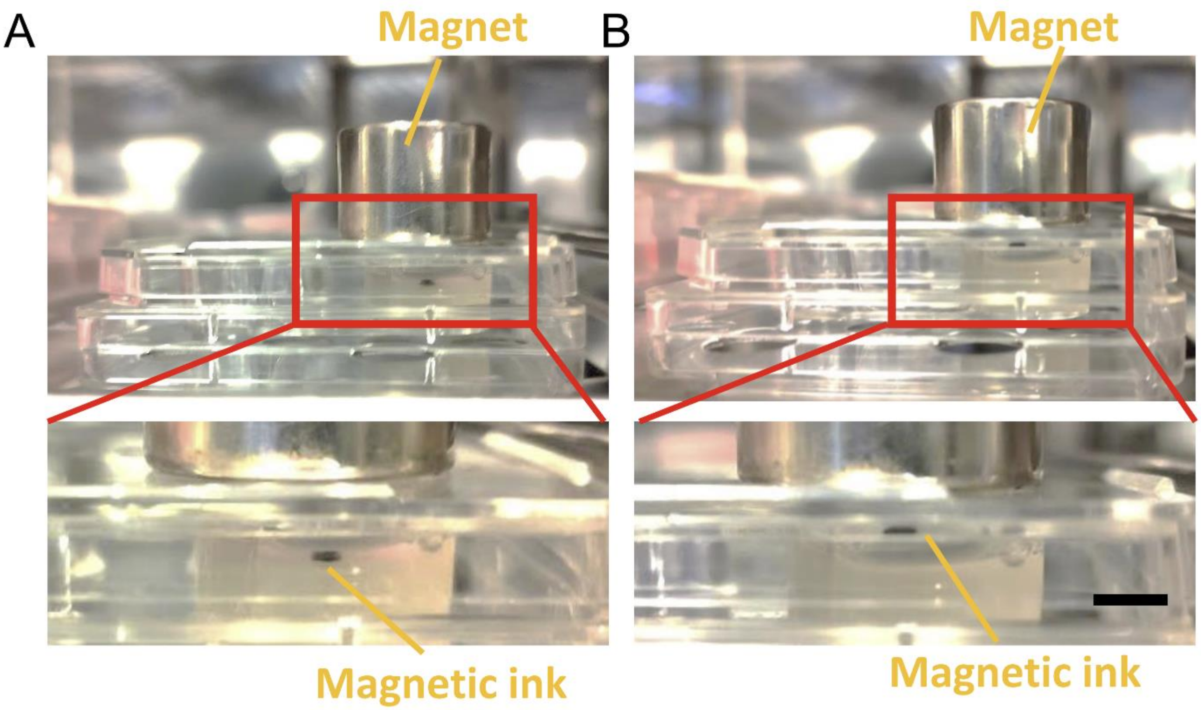

Fig. S8. Magnet aided removal of magnetic ink containing iron oxide particles. The crosslinked bath material with embedded sacrificial 3D branching hydrogels **(A)** before and **(B)** after incubation at 37° C. (Scale bars: 1 cm).

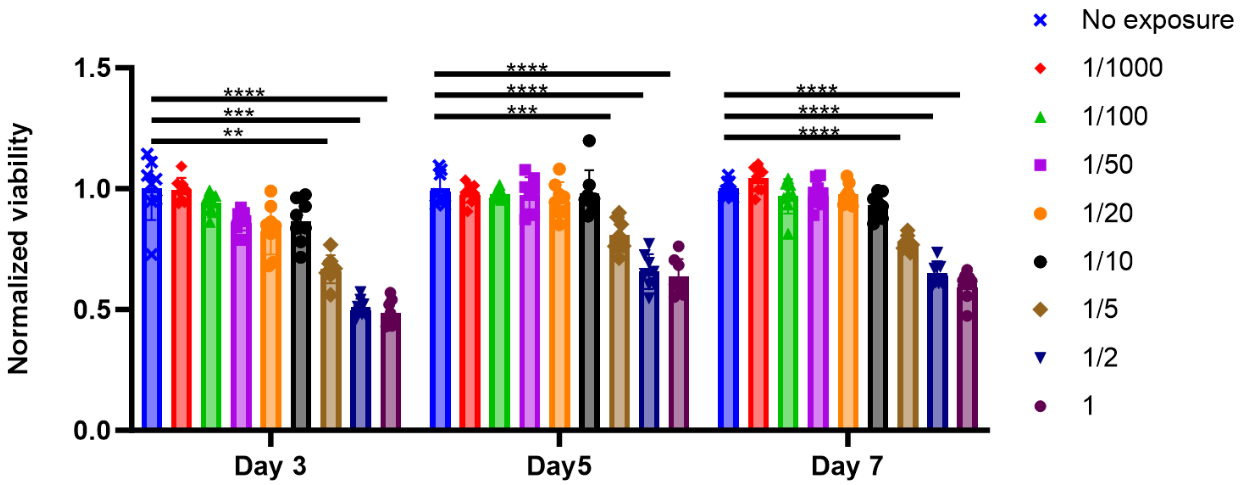

Fig. S9. Cell viability for HUVECs exposed to varying doses of iron oxide particles over a 7-day incubation period. The viability is tested on day 3, day 5 and day 7. Full dose (‘1’) is the concentration equivalent for cells directly exposed to the iron oxide particles within the magnetic ink (1.35 mg/well), and 1/2, 1/5, 1/10, 1/20, 1/50, 1/100, and 1/1000 are dilutions of the full dose. ‘No Exposure’ indicates a control group with no iron oxide particles. (Data were normalized to the average viability of the ‘No Exposure’ groups, shown as mean ± s.d., n = 6 from two independent experiments. $^{**}p < 0.01$, $^{***}p < 0.001$, $^{****}p < 0.0001$ based on two-way ANOVA test.)

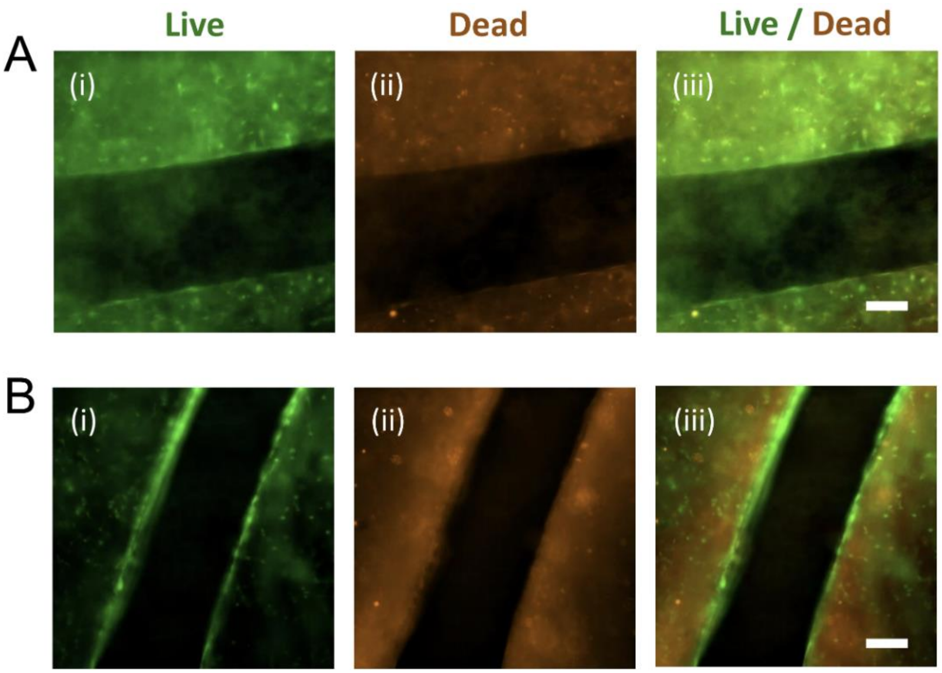

Fig. S10. Viability of embedded Normal Human Lung Fibroblasts (NHLFs) around the channels in GelMA at (A) Day 4 and (B) Day 14. (i) live; (ii) dead; (iii) merging of live and dead images. (Scale bars: 200 μm).

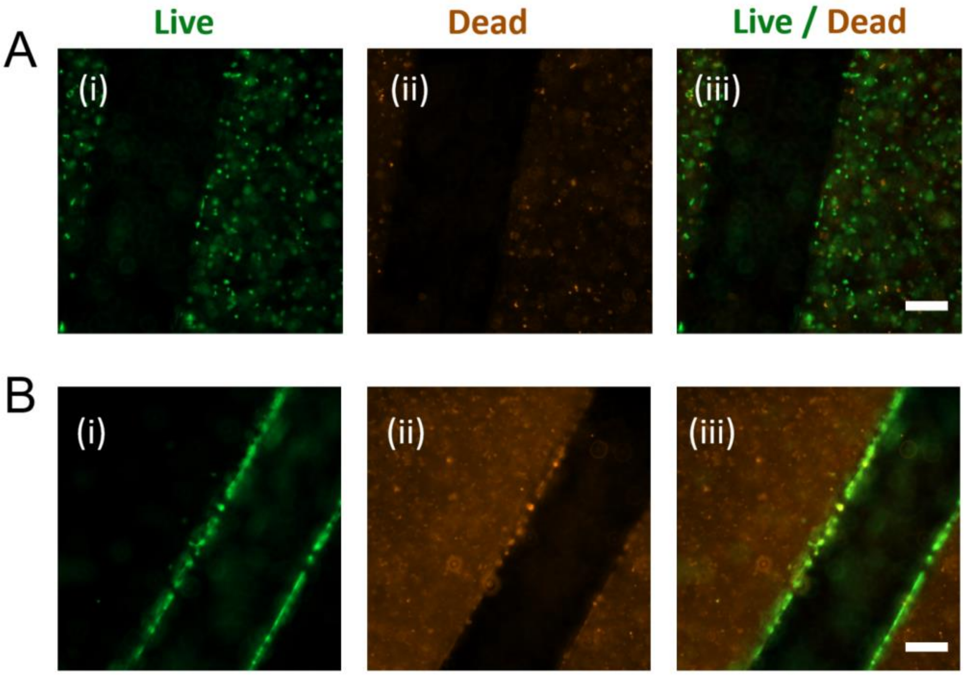

Fig. S11. Viability of embedded HUVECs around the channels in GelMA at (A) Day 4 and (B) Day 14. (i) live; (ii) dead; (iii) merging of live and dead images. (Scale bars: 200 μm).

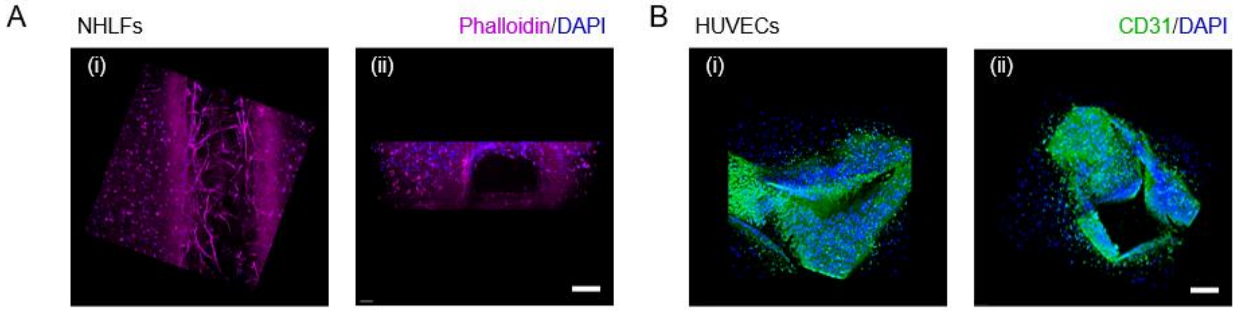

Fig. S12. 3D view of embedded NHLFs or HUVECs around the channels in GelMA. (A) Phalloidin (purple) and DAPI (blue) staining of NHLFs cultured in the perfusable GelMA scaffold. (Scale bars: 200 µm). **(B)** CD31 (green) and DAPI (blue) staining of HUVECs cultured in the perfusable GelMA scaffold. (Scale bars: 200 µm).

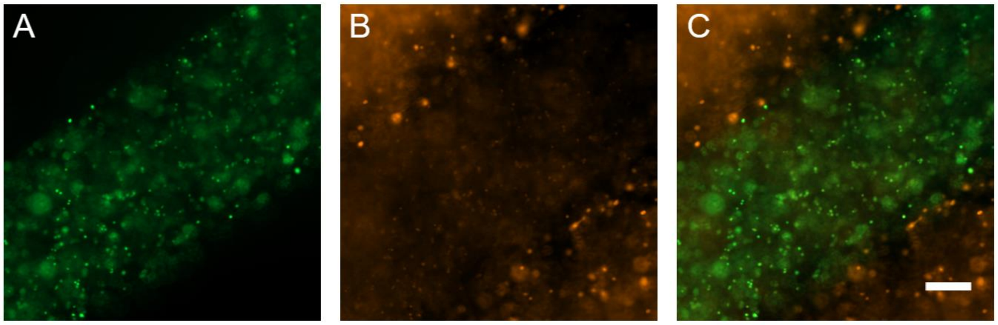

Fig. S13. HUVECs and NHLFs within fibrin during fibrin crosslinking at room temperature. HUVECs were coloured with CellTracker^{TM} Green CMFDA and mixed in gelatin precursor before fabricating the fibrin scaffold. NHLFs were coloured with CellTracker^{TM} Orange CMRA and mixed with fibrin matrix before the fabrication. **(A)** Fluorescent image of 488 nm channel. Green signal indicates the presence of HUVECs. **(B)** Fluorescent image of 555 nm channel. Orange signal indicates the presence of NHLFs. **(C)** Merged image of both channels. (Scale bars: 200 µm).

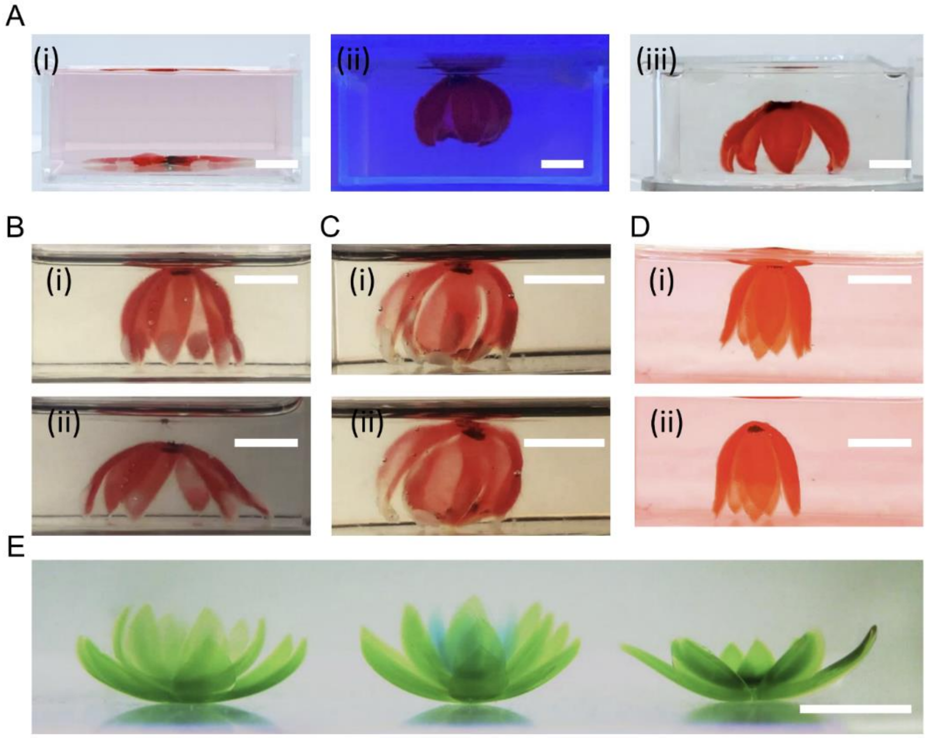

Fig. S14. Fabrication of 3D bioscaffolds using different materials. (A) (i) GelMA before transformation, (ii) during UV crosslinking, (iii) after crosslinking and with magnet removed. **(B)** DexMA, **(C)** HAMA, and **(D)** alginate scaffolds that are (i) transformed and (ii) crosslinked. **(E)** Assembled multilayer transformed alginate scaffolds. (Scale bars: 1 cm).

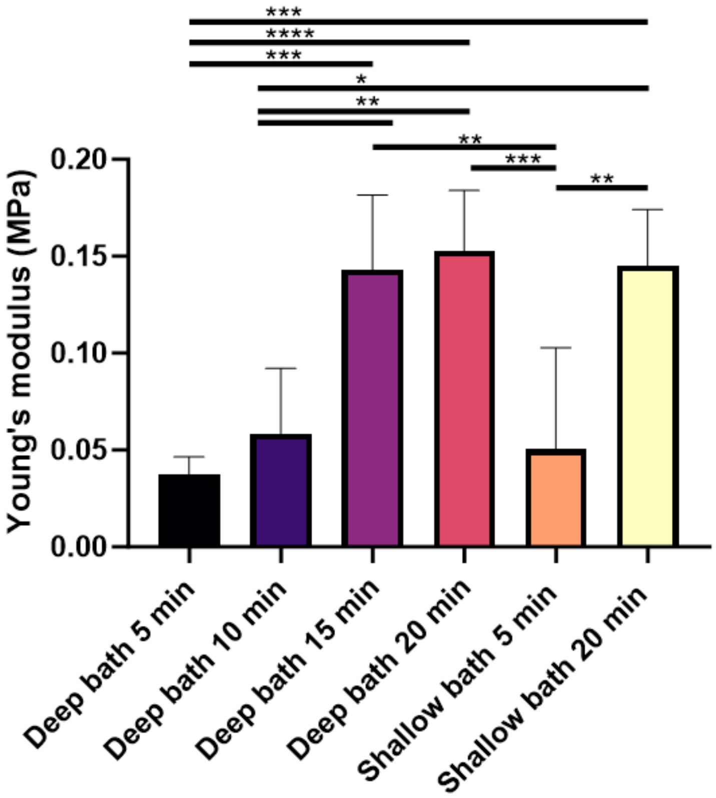

Fig. S15. Young's modulus of GelMA crosslinked under different conditions. The bath was filled with a 2 mM LAP solution to a depth of 15 mm (deep) or 5 mm deep (shallow). The GelMA scaffolds were crosslinked inside the baths for 5 min, 10 min, 15 min, or 20 min. (Data shown as mean± s.d., $n > 4$ from two independent experiments. $*p < 0.1$, $**p < 0.01$, $***p < 0.001$, $****p < 0.0001$ based on one-way ANOVA test.).

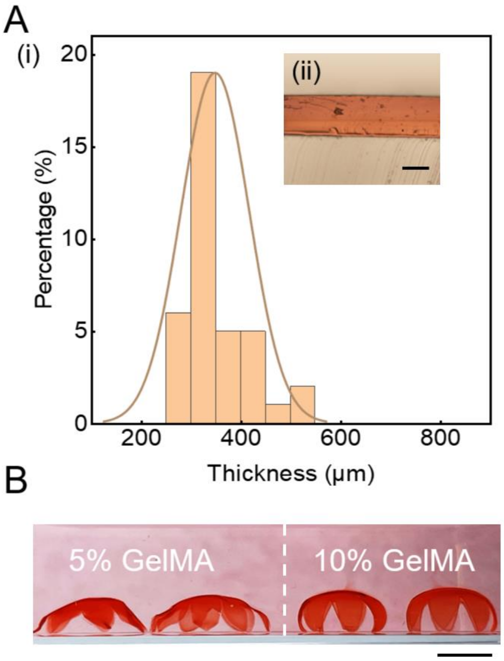

Fig. S16. Freestanding 3D thin-walled bioscaffolds for fabricating biohybrid actuators. (A) (i) The distribution of bioscaffold thickness (n = 38 from N=19 bioscaffolds). (ii) A representative image showing the cross-section of the bioscaffold. (Scale bar: 200 μm). **(B)** Bioscaffolds made of 5% (w/v) GelMA and 10% (w/v) GelMA after gelatin removal. (Scale bars: 1 cm).

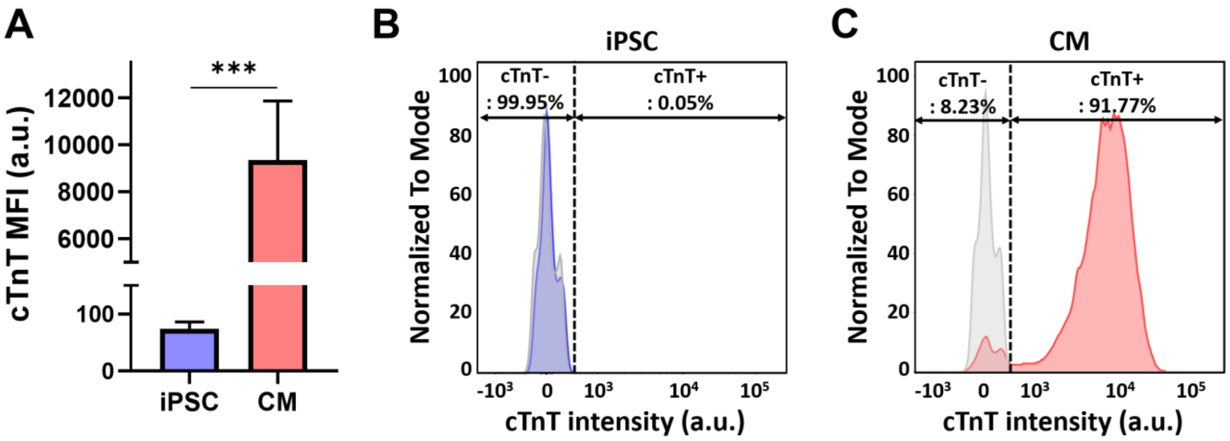

Fig. S17. Flow cytometry analysis of cardiac troponin (cTnT) of induced pluripotent stem cells (iPSCs) and differentiated cardiomyocytes (CM). (A) Mean intensity of cTnT of iPSCs and CM. Results are shown as mean±S.D. from N = 3. **(B)-(C)**. Histogram overlay showing isotype control goat IgG-Alexa 647 (Gray) and cTnT-Alexa 647 of iPSCs (blue) and CM (red). The percentage of cTnT-positive cells (cTnT+) is shown as a mean from N = 3. The two-tailed unpaired Student's t-test was used to analyze the data; ***, $p < 0.001$.

Movie S1. Magnetically driven 4D printing.

Movie S2. Cardiomyocytes triggered contraction and relaxation of the 3D thin-walled bioscaffold.

Movie S3. Top and side view of the moving biohybrid actuator.
